# Supplementary material for: Factors influencing primary care access, utilisation and quality of management for patients living with hypertension in West Africa: a scoping review protocol
Source: BMJ Open. 2024 Jan 23;14(1):e077459. doi: 10.1136/bmjopen-2023-077459 (PMC10824043; doi:10.1136/bmjopen-2023-077459)
Supplement: Author's manuscript [file bmjopen-2023-077459.draft_revisions.pdf]

BMJ Open is committed to open peer review. As part of this commitment we make the peer review history of every article we publish publicly available.

When an article is published we post the peer reviewers' comments and the authors' responses online. We also post the versions of the paper that were used during peer review. These are the versions that the peer review comments apply to.

The versions of the paper that follow are the versions that were submitted during the peer review process. They are not the versions of record or the final published versions. They should not be cited or distributed as the published version of this manuscript.

BMJ Open is an open access journal and the full, final, typeset and author-corrected version of record of the manuscript is available on our site with no access controls, subscription charges or pay-per-view fees (http://bmjopen.bmj.com).

If you have any questions on BMJ Open's open peer review process please email info.bmjopen@bmj.com

BMJ Open

Factors influencing primary care access, utilization, and quality of management for patients living with hypertension in West Africa - A scoping review protocol.

Journal:	*BMJ Open*
Manuscript ID	bmjopen-2023-077459
Article Type:	Protocol
Date Submitted by the Author:	05-Jul-2023

Complete List of Authors:	Amarteyfio, Kezia; Ghana College of Physicians and Surgeons<br>Bondzie, Eugene; Ghana College of Physicians and Surgeons<br>Reichenberger, Veronika; London School of Hygiene & Tropical Medicine Centre of Global Change and Health<br>Agyepong, Irene; Ghana College of Physicians and Surgeons<br>Ansah, Evelyn; Ghana College of Physicians and Surgeons<br>Diarra, Aissa; LASDEL<br>Mirzoev, Tolib; London School of Hygiene & Tropical Medicine Centre of Global Change and Health<br>Perel, Pablo; London School of Hygiene & Tropical Medicine Centre of Global Change and Health<br>Yaogo, Maurice; Universite Catholique de L'Afrique de L'ouest (UCAO) UBB<br>ANTWI, EDWARD; Ghana College of Physicians and Surgeons

Keywords:	Hypertension < CARDIOLOGY, Primary Care < Primary Health Care, Primary Prevention, Blood Pressure

SCHOLARONE™
Manuscripts

Factors influencing primary care access, utilization, and quality of management for patients living with hypertension in West Africa - A scoping review protocol.

- Kezia Amerley Amarteyfio, esikezia@gmail.com[1] / kakosua@rt.gcps.edu.gh

Ghana College of Physicians and Surgeons, 54 Independence Avenue, Ridge Roundabout, Accra, Ghana. Tel no. +2330501155546

- Eugene Paa Kwesi Bondzie, eugenebondzie@gmail.com

Ghana College of Physicians and Surgeons, 54 Independence Avenue, Ridge Roundabout, Accra, Ghana

- Veronica Reichenberger, Veronika.Reichenberger@lshtm.ac.uk

London School of Hygiene and Tropical Medicine

- Irene Agyepong, iagyepong@gcps.edu.gh

Ghana College of Physicians and Surgeons, 54 Independence Avenue, Ridge Roundabout, Accra, Ghana

- Evelyn Korkor Ansah, ansahekdr@yahoo.co.uk

University of Health and Allied Sciences, Ho. Ghana AND Ghana College of Physicians and Surgeons, 54 Independence Avenue, Ridge Roundabout, Accra, Ghana

- Aissa Diarra, Aissa.Diarra@ird.fr

LASDEL, Niamey, Niger

- Tolib Mirzoev, Tolib.Mirzoev@lshtm.ac.uk

London School of Hygiene and Tropical Medicine

- Pablo Perel, Pablo.Perel@lshtm.ac.uk

London School of Hygiene and Tropical Medicine

- Maurice Yaogo, yaogo@hotmail.com

Catholic University of West Africa, Bobodialassou, Burkina Faso

- Edward Antwi, ed_antwi@yahoo.com

Ghana Health Service, Family Health Division AND Ghana College of Physicians and Surgeons, 54 Independence Avenue, Ridge Roundabout, Accra, Ghana

[1] Corresponding Author

For peer review only - http://bmjopen.bmj.com/site/about/guidelines.xhtml

Keywords: Hypertension, Primary care, Context, West Africa , health system

Word count 2675

Abstract

Introduction

Hypertension, one of the most prevalent non-communicable diseases in West Africa, can be well managed with good primary care. This scoping review will explore what is documented in the literature about factors that influence primary care access, utilization, and quality of management for patients living with hypertension in West Africa.

Methods and analysis

The scoping review will employ the approach described by Arksey and O'Malley in 2005. The approach has five stages: (1) formulating the research questions, (2) identifying relevant studies, (3) selecting eligible studies, (4) charting the data and (5) collating, summarizing, and reporting the results. This review will employ the Preferred Reporting Items for Systematic Reviews and Meta-Analysis of Protocols (PRISMA-P) and the PRISMA extension for scoping reviews to present the results.

Databases to be searched are PubMed, Embase, Scopus, Cairn Info, and google scholar from the year 2000 to 2023.

Ethics and dissemination

This review does not require ethics approval. Our dissemination strategy includes peer review publications, policy briefs, and presentations at conferences, stakeholders, and intervention co-production forums.

Strength and limitations of this study

- The paper will be key in identifying areas of needed research in the primary care management of hypertension in West Africa
- This study will also help identify strengths, weaknesses, and areas of challenges in the access, utilization, and management of hypertension in primary care facilities.
- There is a possibility of bias because the review will only include studies in the official languages used in West Africa (English, French, and Portuguese).

For peer review only - http://bmjopen.bmj.com/site/about/guidelines.xhtml

Introduction

Background and extent of the problem

Over the last few decades, non-communicable diseases (NCDs) have become the largest cause of morbidity and mortality worldwide. According to the World Health Organization, in 2019, 41 million out of the 52 million global deaths were due to non-communicable diseases. One of the NCDs responsible for most deaths was cardiovascular diseases including hypertension. Cardiovascular disease accounts for 17.9 million deaths every year[1], most of which are people in LMICs. In 2016, 78% of all NCD deaths, and 85% of premature adult NCD deaths, were in low- and middle-income countries (LMICs)[2]. Research has also found that living in a low socioeconomic area in a high income country (HIC), low or middle income country (LMIC) increases the risk of developing cardiovascular diseases[3].

Hypertension, also known as raised blood pressure, is diagnosed when blood pressure is measured on two different days and the systolic blood pressure readings on both days is greater or equal to 140 mmHg and/or the diastolic blood pressure readings on both days is greater or equal to 90 mmHg[4]. Hypertension is a precursor for various diseases such as ischemic and hemorrhagic stroke, chronic kidney disease, and coronary artery disease[5]. Hypertension is common in West Africa. In 2015, 27% of the population in the African region had high blood pressure[2]. A study on hypertension prevalence, awareness, treatment and control done in seven communities in east and west Africa comprising 3547 participants, showed that a quarter of participants had hypertension, about 40% were unaware, half of those aware were treated, and half of those treated had controlled blood pressure[6]. Another study on the rural-urban difference in the prevalence of hypertension in West Africa showed prevalence of hypertension was 32.6% in males, and 30.0% in females with a lower odd of hypertension in rural compared to urban dwellers.[7] Hypertension prevalence is due to many factors including unhealthy lifestyles, poor health-seeking behavior and poor adherence to medication. All these contribute to uncontrolled cases and poor clinical outcomes even among treated patients with hypertension[8]. Good access to and utilization of adequate quality primary care for people living with hypertension can potentially reduce complications and avoidable hospitalization and mortality[9,10]. Weak and under-resourced health systems and the marginalization of NCD care in LIC make it harder to ensure prevention, early diagnosis, access to and utilization of adequate quality primary care and treatment of patients with hypertension, resulting in the high prevalence. The increase in risk factors such as poor

For peer review only - http://bmjopen.bmj.com/site/about/guidelines.xhtml

dietary habits and physical inactivity that accompany rapid urbanization and related lifestyle changes, also contributes to the high morbidity and mortality associated with hypertension in LMIC[5].

The Economic Community of West Africa states (ECOWAS) sub-region is made up of 15 LMICs who share similar social, economic, and political environments. The countries in the ECOWAS are Benin, Burkina Faso, Cabo Verde, Cote d'Ivoire, The Gambia, Ghana, Guinea, Guinea-Bissau, Liberia, Mali, Niger, Nigeria, Senegal, Sierra Leone, and Togo[11]. A study of 192,441 hypertensive patients in 44 LMIC, including countries in West Africa, showed that only 18%–29.9% of participants received treatment for hypertension, with 5%–10.3% achieving control of their hypertension[12]. Countries in the Caribbean and Latin America did better than countries in Sub Saharan Africa. The study was done to determine the cascade of hypertension care in 44 LMICs – and its variation between countries and population groups[12]. Hypertension control encompasses not just medications but also lifestyle and dietary changes[1]. It is necessary therefore for hypertension control to be addressed not only on the patient and physician level but also on the community, facility, health system and policy levels. To achieve SDG goal 3.4 i.e., to reduce by one-third premature mortality from NCDs through prevention and treatment, there is the need to explore barriers to the control of NCDs in West Africa.

Contextual factors can simply be defined as a range of factors outside of the individual that influence health seeking behaviour. These factors include personal, social, cultural, economic, and political factors that exist in contrasting ways and have differing impacts across population groups[13]. Sociocultural and socioeconomic factors have been documented to influence the management of hypertension. A study by Sommer et al.[3] in LMIC showed that low socioeconomic status was associated with an increased risk of developing cardiovascular diseases. Cultural beliefs and misconceptions also affect patients' approach to the management of hypertension. Patients' belief in spiritual cause of illness and healing[14], as well as the use traditional medicine[15] as opposed to orthodox medicine leads to poor hypertension control. In Ghana for instance, some patients believe there are traditional medicines that can cure hypertension and so combine them with allopathic drugs to treat the disease[16]. These contextual factors influence an individual's decision to seek and maintain treatment for hypertension and are therefore relevant in exploring the barriers to hypertension care at primary care level in West Africa.

For peer review only - http://bmjopen.bmj.com/site/about/guidelines.xhtml

Other factors that influence the management of hypertension are individual, community, primary health care facility and health system factors. Some individual/ patient factors like medication non-adherence, poor lifestyle and dietary choices, lack of awareness and funds to purchase medications leads to poor management of high blood pressure[14,16,17]. Health system challenges such as poor referral systems, inadequate tools and equipment, insufficient funding and lack of sufficient health personnel poses challenges in the access to care and quality of management of hypertension at primary care facilities[16,18,19].

Primary care is defined by the WHO as 'a model of care that supports first-contact, accessible, continuous, comprehensive and coordinated person-focused care'[20]. It is the most basic form of care available to patients at the base of the health care pyramid. It should be accessible to all citizens and be able to provide primary and secondary prevention for common diseases in the community. Primary care is very important because it has been shown to meet the needs of majority of people throughout their lives[20]. With the emergence of NCDs there has been the need to strengthen primary care in the management of hypertension, diabetes and other major NCDs in the West African sub-region.

Rationale

Research into the risk factors, prevalence, and prevention of non-communicable diseases in West Africa has been increasing, although much of the data has not been synthesized into a common thread. There is an urgent need to explore the available data on the barriers and enablers to the control of non-communicable diseases in West Africa. Although a similar scoping review was done on the reasons for poor blood pressure control in eastern Sub-Saharan Africa[21], there is no similar study for Western SSA. Also, the study in eastern SSA did not delve into the contextual factors that iinfluence access, utilization, and quality of primary care for hypertension in West Africa. It focused more on patient, professional, primary healthcare system, and public health policy challenges. To make changes to the current health care for hypertension, it is important to know what has been done so far and the challenges in achieving adequate primary care for hypertension across the various levels in West Africa. For the patients as well as health practitioners, this review will serve to inform understanding of the challenges in the management of hypertension in primary care facilities. The findings of this research will also be key in informing co-production of interventions as well as identifying areas of needed research in the primary care management of hypertension in West Africa.

For peer review only - http://bmjopen.bmj.com/site/about/guidelines.xhtml

Objectives

This review aims to synthesize what is documented in the literature about the factors that hinder and or enable primary care for hypertension at individual, context, health facility and health system levels in West Africa and how and why they work. This includes access, utilization as well as quality of available services.

The specific objectives of this study are to:

1. Describe the factors documented in the literature that hinder and/ or enable management of hypertension at primary care level in West Africa (including access, utilization, and quality of care); and how and why they work.

  1.1. Describe contextual factors (national and global, socio-cultural, socio-economic, political) and analyze how and why they work.

  1.2. Describe individual/ patient factors and analyze how and why they work.

  1.3. Describe health facility and health system factors and analyze how and why they work.

2. To identify relevant gaps in the existing literature and areas of research interest, in the primary care of hypertension in West Africa.

Conceptual framework

A conceptual framework of factors that influence the primary care access, utilization and quality for people living with hypertension in west Africa has been developed to help structure our review as illustrated in Figure 1 below. This framework was adapted from a study on facilitators and barriers to NCD prevention in Pakistan[22] and modified with information from additional studies[8,23].

For peer review only - http://bmjopen.bmj.com/site/about/guidelines.xhtml

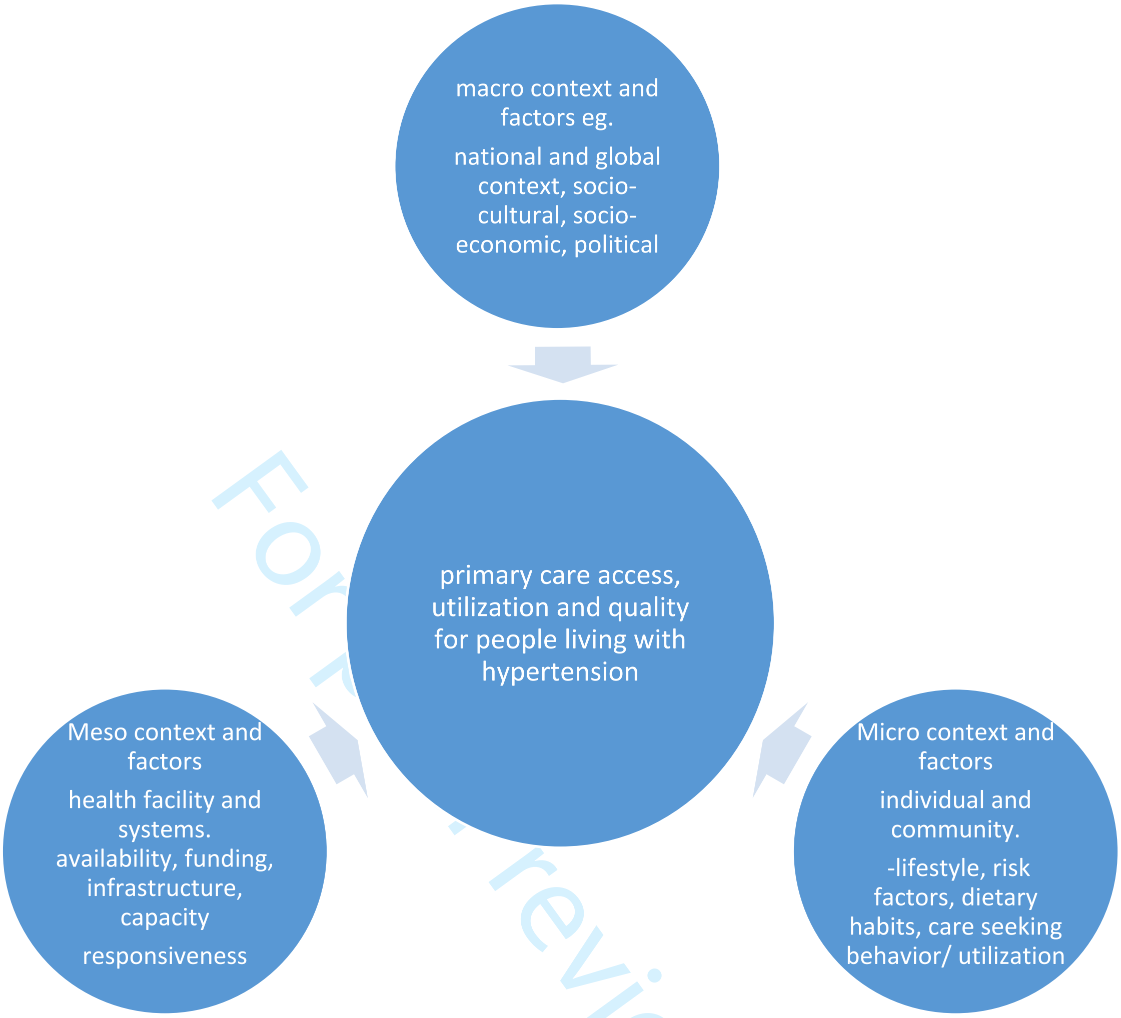

Figure 1-conceptual framework on the factors that influence primary care access, utilization and quality for people living with hypertension

Methods

A scoping review methodology as proposed by Arksey and O'Malley(2005)[24] will be used due to the broad nature of the research question and the uncertainty of the nature and quality of the evidence available to answer the questions. The approach consists of five stages: (1) formulating the research questions, (2) identifying relevant studies, (3) selecting eligible studies, (4) charting the data and (5) collating, summarizing, and reporting the results. Scoping reviews are useful in presenting a broad overview of the data available on a topic and allows one to identify gaps in knowledge and research[25]. It will also help to determine if a systematic review would be relevant in this area of research. The review will apply the PRISMA extension for scoping reviews to present the results[26]. The scoping review also draws inspiration from methodology developed by the Joanna Briggs Institute[27].

For peer review only - http://bmjopen.bmj.com/site/about/guidelines.xhtml

The research question was obtained through discussion with key health system stakeholders as well as researchers in the STOP NCD project, which started implementation in October 2022. The protocol was not registered on Prospero as Prospero currently does not allow for registration of scoping review protocols.

Patient and public involvement

No patients were involved in the development of the review protocol.

Identifying the research question

We used an iterative process, as recommended by Arksey and O'Malley, to develop the research question through consultations with the research team and key health system stakeholders. The overall main research question is defined as:

What are the factors (at the individual, community, health system and context level) that influence access, utilization and primary care management of people living with hypertension in West Africa and how and why do they work?

Identifying relevant studies

Searches will be conducted using PubMed, Embase, Scopus, Cairn Info (primarily for French papers) and google scholar databases for the years 2000 to 2023 with the help of a librarian. This time frame was selected because there has been a surge in the burden of NCDs in sub-Saharan Africa over the past two decades, driven by increasing incidence of cardiovascular risk factors such as unhealthy diets, reduced physical activity, hypertension, obesity, diabetes, dyslipidemia, and air pollution[28].The key concepts from our research question and alternative search terms (see Appendix 1) will be combined using the Boolean terms AND, NOT and OR. Excel power query will be used to generate all combinations of the key search terms to perform an exhaustive search. An example of the search strategy using PubMed is illustrated in Appendix 2. The search strategy will be adapted as appropriate for each of the search databases. Both published and grey literature that are relevant will be included. Other literature will be found using the software 'research rabbit' by inputting relevant papers. Some literature will also be found by searching the reference list and citations of key articles used in the review. Relevant websites such as the WHO website will be accessed for grey

For peer review only - http://bmjopen.bmj.com/site/about/guidelines.xhtml

literature and included in the study. The search results will be exported into a reference manager software (e.g. endnote) to allow for easy referencing and removal of duplicates.

Study selection

The studies found will be screened against the pre-specified eligibility criteria and study selection and will be done by two independent researchers. The studies will first be examined with their titles and abstract, and studies that are obviously irrelevant will be removed. Relevant studies will be retrieved and examined fully to determine eligibility. Any disagreements will be resolved through discussion and where necessary, involvement of a third independent researcher. There will be an initial pilot selection of a few papers to test the search eligibility criteria prior to the start of the main selection. This will be done by two independent reviewers to ensure reliability. The final results of the search will be reported in full in the final report and presented in a PRISMA flow diagram[29](Appendix 4). The reason for excluding any studies will be documented and included in our final report. Additionally, a description of the PCCS framework which stands for population, concept, context, and study elements is outlined below to guide the screening and identification of relevant studies.

Table 1

Population	Concept	Context	Study
Adults 18 years or older with systemic hypertension in West Africa	Hindering and enabling factors that influence first level of care for hypertension	Macro (National and global level factors)<br>Meso (Health System factors including Primary care facilities<br>Micro (Community factors)	Qualitative, quantitative, meta-analysis, social science case studies and case series

Inclusion criteria

For peer review only - http://bmjopen.bmj.com/site/about/guidelines.xhtml

Relevant papers written between 2000 to 2023 with participants over the age of 18 years will be included.

Articles, systematic reviews, observational studies, and reports that include information on the relevant factors that influence primary care management of hypertension in West Africa will be included in the paper.

Papers written in English, French and Portuguese (Guinea-Bissau and Cape Verde) will be included.

Exclusion criteria

Clinical case series/ case reports, short communications, and conference proceedings will be excluded.

Papers on gestational hypertension and pre-eclampsia will be excluded.

Data Charting

The data that will be charted will be entered onto a 'data charting form' using the database program Excel. The data charting form will include the name of the author, study type, year of publication, study design, study population, sample size, study objectives, type of evidence source (i.e., primary study, review, website etc.) key findings, comments, and limitations. An illustration is shown in Appendix 3. The two independent reviewers will test the data charting form on a few studies to ensure validity. Any disagreement will be settled by consensus or through the involvement of a third independent reviewer.

Collating, summarizing, and reporting the results.

The available evidence on the factors that influence primary care for hypertensive patients will be described and summarized under themes with the following headings: individual, contextual, health systems/health facility and gaps in the research. Individual factors include lifestyle, dietary habits and risk factors that impede or encourage management of hypertension. These factors include patient's adherence to medications and recommended lifestyle changes and barriers to achieving hypertension control. Contextual factors will be explored at macro (national and global levels), meso (sub-national including health system and facility levels), and micro (community and household levels). At the macro level this is summarized as the socio-cultural, socio-economic, and political environment that

For peer review only

hinder/enable primary care management of hypertension management. At the meso level, the health system and health facility factors are the issues that enable/prevent primary care management for patients living with hypertension. The data collected from the included studies will be tabulated and summarized narratively.

Additional limits

One of the limitations envisaged in this study would be the language barrier in accessing papers from French-speaking countries. We plan to get around this problem by asking colleague researchers in Niger and Burkina Faso to assist in the search. Google translation together with the employment of a bilingual secretary will help address language as a barrier.

Conclusion

Hypertension is a major preventable non-communicable disease that needs to be addressed urgently in West Africa. There is the need to know what has been done so far in identifying the factors responsible for the poor hypertension control in west Africa. This scoping review will describe the available evidence and identify the gaps and approaches to inform policy. This review will also generate evidence to guide research and assist development organisations, governments and other stakeholders to identify priority areas for NCD strategies.

Ethics and dissemination

This review does not require ethics approval. Our dissemination strategy includes peer review publication, policy brief, presentation at conferences and relevant stakeholders.

Acknowledgement

We thank colleagues on the Stop-NCD context analysis team for their comments and feedback on the earlier drafts of this protocol.

Funding

This work received funding from NIHR (National Institute for health research) global Health Research centers project: Research and Institutional capacity Strengthening in NCDs. Grant number NIHR203246 – Global Health Research Centre on Strengthening of Capacity for NCD control in West Africa (Stop-NCD).

Competing interests

For peer review only

None declared.

Data statement

No new data generated or analyzed

Author Statement

KAA drafted the research question, study design and methods and final editing, drafting and submission of the protocol. EPKB, VR, IA, EKA, AD, TM,PP, MY and EA aided in developing the research question and study methods, contributed meaningfully to the drafting and editing, and approved the final manuscript.

References

1. Non communicable diseases. Accessed March 15, 2023. https://www.who.int/news-room/fact-sheets/detail/noncommunicable-diseases
2. World Health Organization. *Noncommunicable Diseases Country Profiles 2018*. World Health Organization; 2018. Accessed March 14, 2023. https://apps.who.int/iris/handle/10665/274512
3. Sommer I, Griebler U, Mahlknecht P, et al. Socioeconomic inequalities in non-communicable diseases and their risk factors: an overview of systematic reviews. *BMC Public Health*. 2015;15:914. doi:10.1186/s12889-015-2227-y
4. Hypertension. Accessed March 14, 2023. https://www.who.int/news-room/fact-sheets/detail/hypertension
5. A GLOBAL BRIEF ON HYPERTENSION WHO.pdf.
6. Okello S, Muhihi A, Mohamed SF, et al. Hypertension prevalence, awareness, treatment, and control and predicted 10-year CVD risk: a cross-sectional study of seven communities in East and West Africa (SevenCEWA). *BMC Public Health*. 2020;20(1):1706. doi:10.1186/s12889-020-09829-5
7. Sani RN, Connelly PJ, Toft M, et al. Rural-urban difference in the prevalence of hypertension in West Africa: a systematic review and meta-analysis. *J Hum Hypertens*. Published online April 16, 2022:1-13. doi:10.1038/s41371-022-00688-8
8. Koduah A, Nonvignon J, Colson A, et al. Health systems, population and patient challenges for achieving universal health coverage for hypertension in Ghana. *Health Policy and Planning*. 2021;36(9):1451-1458. doi:10.1093/heapol/czab088
9. Walker RL, Chen G, McAlister FA, et al. Hospitalization for Uncomplicated Hypertension: An Ambulatory Care Sensitive Condition. *Canadian Journal of Cardiology*. 2013;29(11):1462-1469. doi:10.1016/j.cjca.2013.05.002

For peer review only

For peer review only - http://bmjopen.bmj.com/site/about/guidelines.xhtml

10. Kuecker CM, Kashyap A, Seckel E. Implementation of a Protocol to Manage Patients at Risk for Hospitalization Due to an Ambulatory Care Sensitive Condition. *Fed Pract*. 2020;37(8):380-383. doi:10.12788/fp.0030

11. Economic Community of West African States (ECOWAS). United States Trade Representative. Accessed June 12, 2023. http://ustr.gov/countries-regions/africa/regional-economic-communities-rec/economic-community-west-african-states

12. Geldsetzer P, Manne-Goehler J, Marcus ME, et al. The state of hypertension care in 44 low-income and middle-income countries: a cross-sectional study of nationally representative individual-level data from 1·1 million adults. *Lancet*. 2019;394(10199):652-662. doi:10.1016/S0140-6736(19)30955-9

13. Glossary. Accessed April 26, 2023. https://www.australiancurriculum.edu.au/f-10-curriculum/health-and-physical-education/glossary/

14. Atinga RA, Yarney L, Gavu NM. Factors influencing long-term medication non-adherence among diabetes and hypertensive patients in Ghana: A qualitative investigation. *PLOS ONE*. 2018;13(3):e0193995. doi:10.1371/journal.pone.0193995

15. Laar AK, Adler AJ, Kotoh AM, et al. Health system challenges to hypertension and related non-communicable diseases prevention and treatment: perspectives from Ghanaian stakeholders. *BMC Health Services Research*. 2019;19(1):693. doi:10.1186/s12913-019-4571-6

16. Nyaaba GN, Masana L, de-Graft Aikins A, Beune E, Agyemang C. Factors hindering hypertension control: perspectives of front-line health professionals in rural Ghana. *Public Health*. 2020;181:16-23. doi:10.1016/j.puhe.2019.11.007

17. Buabeng KO. Unaffordable drug prices: the major cause of non-compliance with hypertension medication in Ghana. Published online 2004.

18. Vialle-Valentin CE, Serumaga B, Wagner AK, Ross-Degnan D. Evidence on access to medicines for chronic diseases from household surveys in five low- and middle-income countries. *Health Policy Plan*. 2015;30(8):1044-1052. doi:10.1093/heapol/czu107

19. Iwelunmor J, Blackstone S, Gyamfi J, et al. A Concept Mapping Study of Physicians' Perceptions of Factors Influencing Management and Control of Hypertension in Sub-Saharan Africa. *International Journal of Hypertension*. 2015;2015:e412804. doi:10.1155/2015/412804

20. Primary care. Accessed April 27, 2023. https://www.who.int/teams/integrated-health-services/clinical-services-and-systems/primary-care

21. Link to external site this link will open in a new window. Reasons for poor blood pressure control in Eastern Sub-Saharan Africa: looking into 4P's (primary care, professional, patient, and public health policy) for improving blood pressure control: a scoping review. Published online 2021:1-15. doi:10.1186/s12872-021-01934-6

22. Facilitators and barriers to NCD prevention in Pakistanis–invincibility or inevitability: a qualitative research study | BMC Research Notes | Full Text. Accessed May 5, 2023. https://bmcresnotes.biomedcentral.com/articles/10.1186/s13104-016-2087-2

For peer review only - http://bmjopen.bmj.com/site/about/guidelines.xhtml

23. Sorato MM, Davari M, Kebriaeezadeh A, Sarrafzadegan N, Shibru T, Fatemi B. Reasons for poor blood pressure control in Eastern Sub-Saharan Africa: looking into 4P's (primary care, professional, patient, and public health policy) for improving blood pressure control: a scoping review. *BMC Cardiovascular Disorders*. 2021;21(1):123. doi:10.1186/s12872-021-01934-6

24. Arksey H, O'Malley L. Scoping Studies: Towards a Methodological Framework. *International Journal of Social Research Methodology: Theory & Practice*. 2005;8:19-32. doi:10.1080/1364557032000119616

25. Tricco AC, Lillie E, Zarin W, et al. A scoping review on the conduct and reporting of scoping reviews. *BMC Medical Research Methodology*. 2016;16(1):15. doi:10.1186/s12874-016-0116-4

26. Tricco AC, Lillie E, Zarin W, et al. PRISMA Extension for Scoping Reviews (PRISMA-ScR): Checklist and Explanation. *Ann Intern Med*. 2018;169(7):467-473. doi:10.7326/M18-0850

27. Chapter 11: Scoping reviews. In: *JBI Manual for Evidence Synthesis*. JBI; 2020. doi:10.46658/JBIMES-20-12

28. Bigna JJ, Noubiap JJ. The rising burden of non-communicable diseases in sub-Saharan Africa. *The Lancet Global Health*. 2019;7(10):e1295-e1296. doi:10.1016/S2214-109X(19)30370-5

29. Page MJ, McKenzie JE, Bossuyt PM, et al. The PRISMA 2020 statement: an updated guideline for reporting systematic reviews. *BMJ*. 2021;372:n71. doi:10.1136/bmj.n71

For peer review only

Appendices

Appendix 1- Key concepts and alternative terms.

Search concepts	Alternatives terms
1. Factors	Socio-cultural, socio-economic, social, societal, health systems, individual, patient, community, political
2. Influence	Hinder, enable, promote, threaten, impact, control, motivate, affect,
3.Primary care	Screening, medications, counselling, patient education, prescription, outpatient visits, lifestyle, access, utilization, quality of care

For peer review only - http://bmjopen.bmj.com/site/about/guidelines.xhtml

4. Hypertension	High blood pressure, raised blood pressure, increased blood pressure, hypertensive disease, hypertension diagnosis, hypertension treatment, hypertension control, hypertension cascade, NCD management
5. West Africa	Ghana, Niger, Burkina Faso, Benin, Cape Verde, Côte D'Ivoire, Gambia, Guinea, Guinea-Bissau, Liberia, Mali, Mauritania, Nigeria, Senegal, Sierra Leone, Togo

Appendix 2- search strategy for Pubmed

Filters Year 2000-2023	(("statistics and numerical data"[MeSH Subheading] OR ("statistics"[All Fields] AND "numerical"[All Fields] AND "data"[All Fields]) OR "statistics and numerical data"[All Fields] OR "utilization"[All Fields] OR "utilisation"[All Fields] OR "utilisations"[All Fields] OR "utilise"[All Fields] OR "utilised"[All Fields] OR "utilises"[All Fields] OR "utilising"[All Fields] OR "utilities"[All Fields] OR "utility"[All Fields] OR "utilizations"[All Fields] OR "utilize"[All Fields] OR "utilized"[All Fields] OR "utilizer"[All Fields] OR "utilizers"[All Fields] OR "utilizes"[All Fields] OR "utilizing"[All Fields]) AND "primary health care"[All Fields] AND (("Socio-cultural"[All Fields] OR ("socioeconomic factors"[MeSH Terms] OR ("socioeconomic"[All Fields] AND "factors"[All Fields]) OR "socioeconomic factors"[All Fields] OR "socioeconomics"[All Fields] OR "socioeconomic"[All Fields] OR "socioeconomical"[All Fields] OR "socioeconomically"[All Fields]) OR ("individual s"[All Fields] OR "individualisation"[All Fields] OR "individualise"[All Fields] OR "individualised"[All Fields] OR "individualising"[All Fields] OR "individualism"[All Fields] OR "individualisms"[All Fields]	30

For peer review only - http://bmjopen.bmj.com/site/about/guidelines.xhtml

For peer review only

	OR "individualities"[All Fields] OR "individuality"[MeSH Terms] OR "individuality"[All Fields] OR "individualization"[All Fields] OR "individualize"[All Fields] OR "individualized"[All Fields] OR "individualizes"[All Fields] OR "individualizing"[All Fields] OR "individually"[All Fields] OR "individuals"[All Fields] OR "individuate"[All Fields] OR "individuated"[All Fields] OR "individuates"[All Fields] OR "individuating"[All Fields] OR "individuation"[MeSH Terms] OR "individuation"[All Fields] OR "individuations"[All Fields] OR "persons"[MeSH Terms] OR "persons"[All Fields] OR "individual"[All Fields]) OR ("communal"[All Fields] OR "communalism"[All Fields] OR "communalities"[All Fields] OR "communality"[All Fields] OR "communally"[All Fields] OR "commune"[All Fields] OR "communes"[All Fields] OR "community s"[All Fields] OR "communitys"[All Fields] OR "residence characteristics"[MeSH Terms] OR ("residence"[All Fields] AND "characteristics"[All Fields]) OR "residence characteristics"[All Fields] OR "communities"[All Fields] OR "community"[All Fields]) OR "health system"[All Fields]) AND ("hypertense"[All Fields] OR "hypertension"[MeSH Terms] OR "hypertension"[All Fields] OR "hypertension s"[All Fields] OR "hypertensions"[All Fields] OR "hypertensive"[All Fields] OR "hypertensive s"[All Fields] OR "hypertensives"[All Fields] OR "High blood pressure"[All Fields] OR "raised blood pressure"[All Fields] OR "increased blood pressure"[All Fields] OR "hypertensive disease"[All Fields])) AND ("nigeria"[MeSH Terms] OR "nigeria"[All Fields] OR "nigeria s"[All Fields])) AND (2000:2023[pdat])	

Appendix 3- data charting form

Autho r	Type of	Study desig	Study populatio	Sampl e size	objective s	Key finding	type of evidenc	Comment /

For peer review only - http://bmjopen.bmj.com/site/about/guidelines.xhtml

(name and year)	paper	n	n			s	e source	limitations

Appendix 4- PRISMA 2020 FLOW diagram for systematic reviews

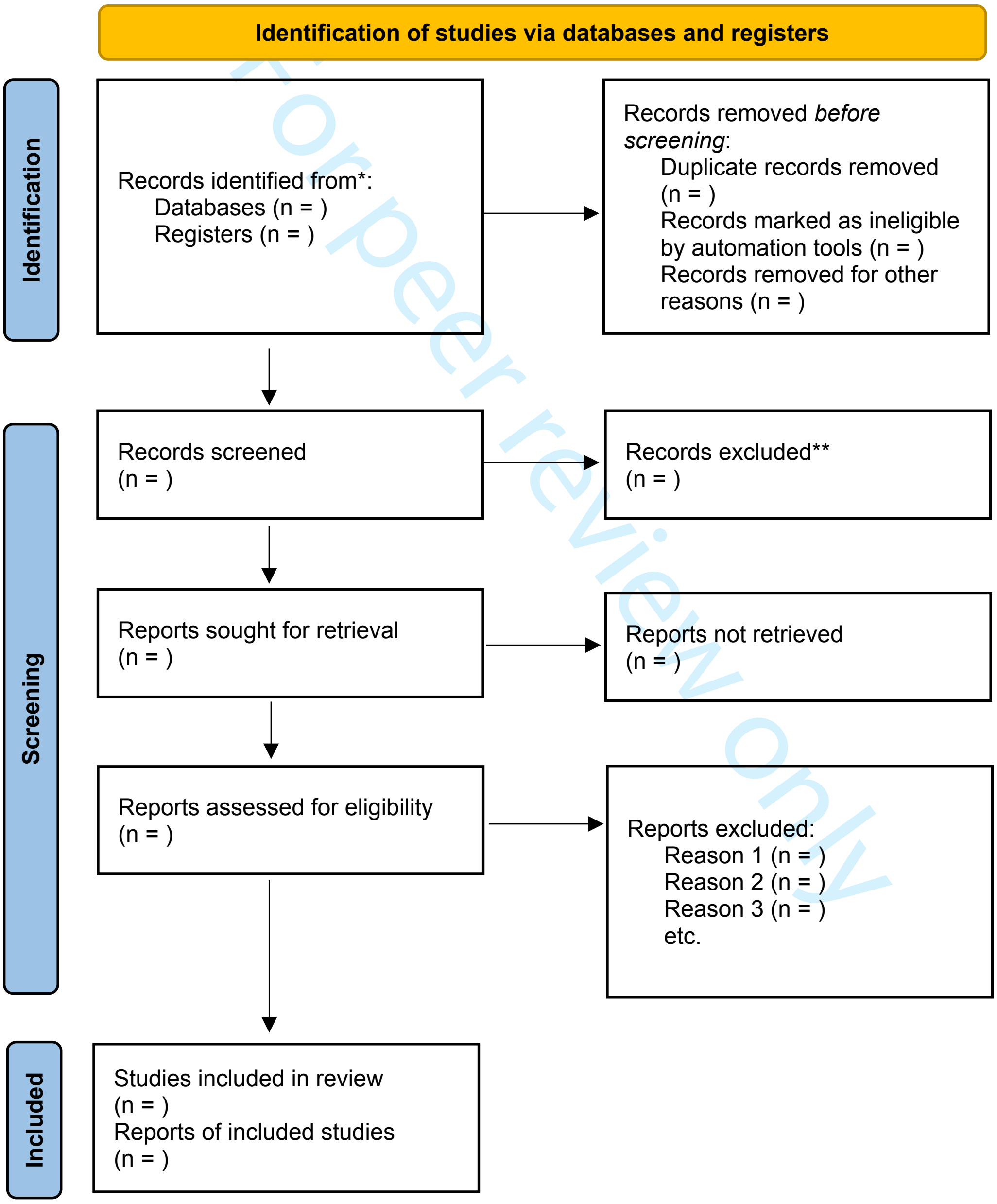

*Consider, if feasible to do so, reporting the number of records identified from each database or register searched (rather than the total number across all databases/registers).

**If automation tools were used, indicate how many records were excluded by a human and how many were excluded by automation tools.

For peer review only - http://bmjopen.bmj.com/site/about/guidelines.xhtml

Appendix 5- Flow chart for the inclusion and exclusion of papers

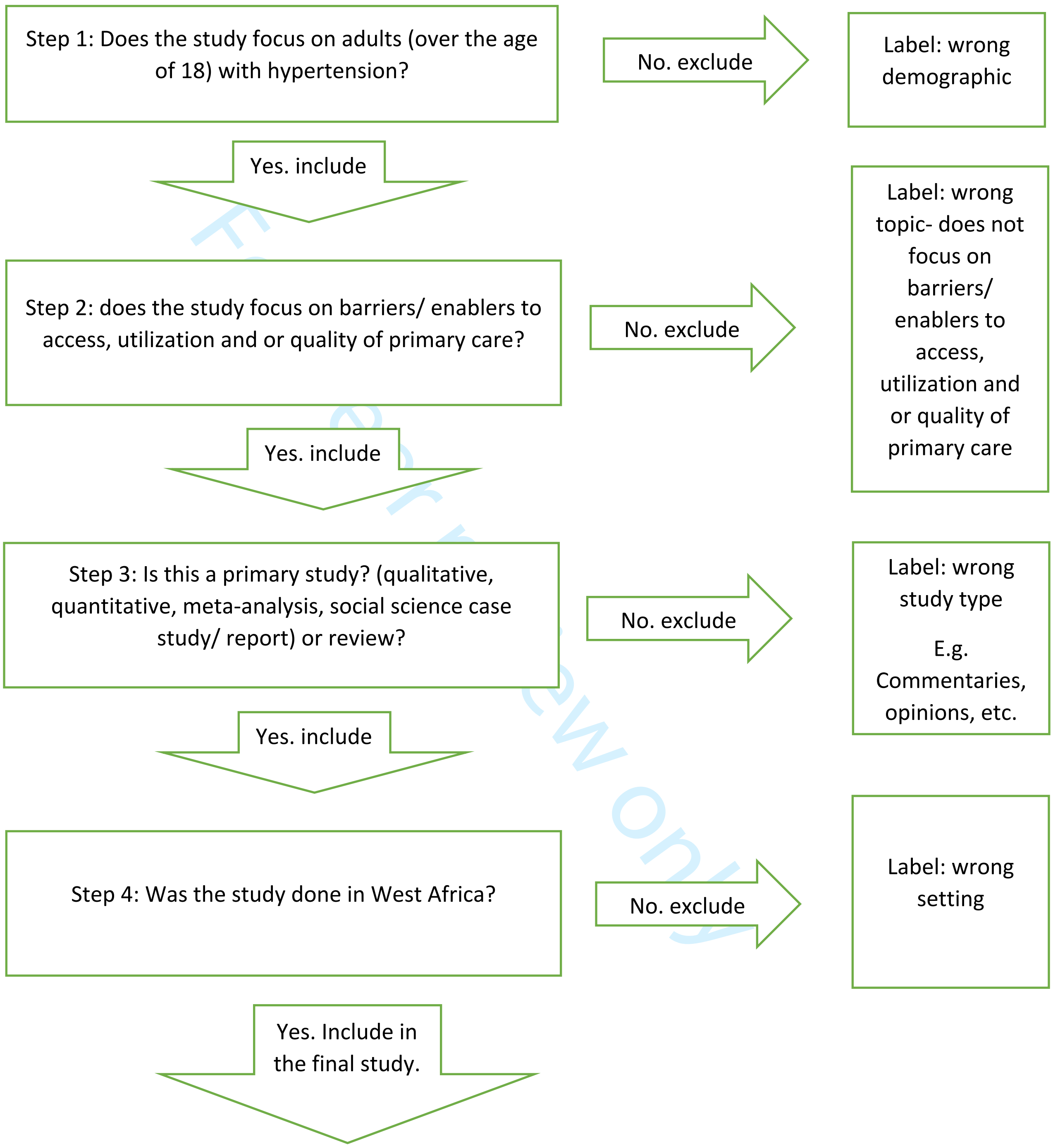

For peer review only - http://bmjopen.bmj.com/site/about/guidelines.xhtml

Preferred Reporting Items for Systematic reviews and Meta-Analyses extension for Scoping Reviews (PRISMA-ScR) Checklist

SECTION	ITEM	PRISMA-ScR CHECKLIST ITEM	REPORTED ON PAGE #
**TITLE**			
Title	1	Identify the report as a scoping review.	
**ABSTRACT**			
Structured summary	2	Provide a structured summary that includes (as applicable): background, objectives, eligibility criteria, sources of evidence, charting methods, results, and conclusions that relate to the review questions and objectives.	
**INTRODUCTION**			
Rationale	3	Describe the rationale for the review in the context of what is already known. Explain why the review questions/objectives lend themselves to a scoping review approach.	
Objectives	4	Provide an explicit statement of the questions and objectives being addressed with reference to their key elements (e.g., population or participants, concepts, and context) or other relevant key elements used to conceptualize the review questions and/or objectives.	
**METHODS**			
Protocol and registration	5	Indicate whether a review protocol exists; state if and where it can be accessed (e.g., a Web address); and if available, provide registration information, including the registration number.	
Eligibility criteria	6	Specify characteristics of the sources of evidence used as eligibility criteria (e.g., years considered, language, and publication status), and provide a rationale.	
Information sources*	7	Describe all information sources in the search (e.g., databases with dates of coverage and contact with authors to identify additional sources), as well as the date the most recent search was executed.	
Search	8	Present the full electronic search strategy for at least 1 database, including any limits used, such that it could be repeated.	
Selection of sources of evidence†	9	State the process for selecting sources of evidence (i.e., screening and eligibility) included in the scoping review.	
Data charting process‡	10	Describe the methods of charting data from the included sources of evidence (e.g., calibrated forms or forms that have been tested by the team before their use, and whether data charting was done independently or in duplicate) and any processes for obtaining and confirming data from investigators.	
Data items	11	List and define all variables for which data were sought and any assumptions and simplifications made.	
Critical appraisal of individual sources of evidence§	12	If done, provide a rationale for conducting a critical appraisal of included sources of evidence; describe the methods used and how this information was used in any data synthesis (if appropriate).	
Synthesis of results	13	Describe the methods of handling and summarizing the data that were charted.	

St. Michael's
Inspired Care.
Inspiring Science.

For peer review only - http://bmjopen.bmj.com/site/about/guidelines.xhtml

SECTION	ITEM	PRISMA-ScR CHECKLIST ITEM	REPORTED ON PAGE #
**RESULTS**			
Selection of sources of evidence	14	Give numbers of sources of evidence screened, assessed for eligibility, and included in the review, with reasons for exclusions at each stage, ideally using a flow diagram.	
Characteristics of sources of evidence	15	For each source of evidence, present characteristics for which data were charted and provide the citations.	
Critical appraisal within sources of evidence	16	If done, present data on critical appraisal of included sources of evidence (see item 12).	
Results of individual sources of evidence	17	For each included source of evidence, present the relevant data that were charted that relate to the review questions and objectives.	
Synthesis of results	18	Summarize and/or present the charting results as they relate to the review questions and objectives.	
**DISCUSSION**			
Summary of evidence	19	Summarize the main results (including an overview of concepts, themes, and types of evidence available), link to the review questions and objectives, and consider the relevance to key groups.	
Limitations	20	Discuss the limitations of the scoping review process.	
Conclusions	21	Provide a general interpretation of the results with respect to the review questions and objectives, as well as potential implications and/or next steps.	
**FUNDING**			
Funding	22	Describe sources of funding for the included sources of evidence, as well as sources of funding for the scoping review. Describe the role of the funders of the scoping review.	

JBI = Joanna Briggs Institute; PRISMA-ScR = Preferred Reporting Items for Systematic reviews and Meta-Analyses extension for Scoping Reviews.

* Where *sources of evidence* (see second footnote) are compiled from, such as bibliographic databases, social media platforms, and Web sites.

† A more inclusive/heterogeneous term used to account for the different types of evidence or data sources (e.g., quantitative and/or qualitative research, expert opinion, and policy documents) that may be eligible in a scoping review as opposed to only studies. This is not to be confused with *information sources* (see first footnote).

‡ The frameworks by Arksey and O'Malley (6) and Levac and colleagues (7) and the JBI guidance (4, 5) refer to the process of data extraction in a scoping review as data charting.

§ The process of systematically examining research evidence to assess its validity, results, and relevance before using it to inform a decision. This term is used for items 12 and 19 instead of "risk of bias" (which is more applicable to systematic reviews of interventions) to include and acknowledge the various sources of evidence that may be used in a scoping review (e.g., quantitative and/or qualitative research, expert opinion, and policy document).

From: Tricco AC, Lillie E, Zarin W, O'Brien KK, Colquhoun H, Levac D, et al. PRISMA Extension for Scoping Reviews (PRISMAScR): Checklist and Explanation. Ann Intern Med. 2018;169:467–473. doi: 10.7326/M18-0850.

For peer review only - http://bmjopen.bmj.com/site/about/guidelines.xhtml

BMJ Open

Factors influencing primary care access, utilization, and quality of management for patients living with hypertension in West Africa: a scoping review protocol

Journal:	*BMJ Open*
Manuscript ID	bmjopen-2023-077459.R1
Article Type:	Protocol
Date Submitted by the Author:	26-Dec-2023

Complete List of Authors:	Amarteyfio, Kezia; Ghana College of Physicians and Surgeons<br>Bondzie, Eugene; Ghana College of Physicians and Surgeons<br>Reichenberger, Veronika; London School of Hygiene & Tropical Medicine Centre of Global Change and Health<br>Agyepong, Irene; Ghana College of Physicians and Surgeons<br>Ansah, Evelyn; Ghana College of Physicians and Surgeons<br>Diarra, Aissa; LASDEL<br>Mirzoev, Tolib; London School of Hygiene & Tropical Medicine Centre of Global Change and Health<br>Perel, Pablo; London School of Hygiene & Tropical Medicine Centre of Global Change and Health<br>Yaogo, Maurice; Universite Catholique de L'Afrique de L'ouest (UCAO) UBB<br>ANTWI, EDWARD; Ghana College of Physicians and Surgeons

**Primary Subject Heading**:	Qualitative research
Secondary Subject Heading:	Global health, Public health
Keywords:	Hypertension < CARDIOLOGY, Primary Care < Primary Health Care, Primary Prevention, Blood Pressure

SCHOLARONE™
Manuscripts

Factors influencing primary care access, utilization, and quality of management for patients living with hypertension in West Africa: a scoping review protocol

- Kezia Amerley Amarteyfio, esikezia@gmail.com[1] kakosua@rt.gcps.edu.gh

Ghana College of Physicians and Surgeons, 54 Independence Avenue, Ridge Roundabout, Accra, Ghana. Tel no. +2330501155546

- Eugene Paa Kofi Bondzie, eugenebondzie@gmail.com

Ghana College of Physicians and Surgeons, 54 Independence Avenue, Ridge Roundabout, Accra, Ghana

- Veronica Reichenberger, Veronika.Reichenberger@lshtm.ac.uk

London School of Hygiene and Tropical Medicine

- Irene Agyepong, iagyepong@gcps.edu.gh

Ghana College of Physicians and Surgeons, 54 Independence Avenue, Ridge Roundabout, Accra, Ghana

- Evelyn Korkor Ansah, ansahekdr@yahoo.co.uk

University of Health and Allied Sciences, Ho. Ghana AND Ghana College of Physicians and Surgeons, 54 Independence Avenue, Ridge Roundabout, Accra, Ghana

- Aissa Diarra, Aissa.Diarra@ird.fr

LASDEL, Niamey, Niger

- Tolib Mirzoev, Tolib.Mirzoev@lshtm.ac.uk

London School of Hygiene and Tropical Medicine

- Pablo Perel, Pablo.Perel@lshtm.ac.uk

London School of Hygiene and Tropical Medicine

- Maurice Yaogo, yaogo@hotmail.com

Catholic University of West Africa, Bobodialassou, Burkina Faso

- Edward Antwi, ed_antwi@yahoo.com

Ghana Health Service, Family Health Division AND Ghana College of Physicians and Surgeons, 54 Independence Avenue, Ridge Roundabout, Accra, Ghana

[1] Corresponding Author

For peer review only - http://bmjopen.bmj.com/site/about/guidelines.xhtml

Keywords: Hypertension, Primary care, Access, Quality, Utilization, Context, West Africa, health system

Word count 2589

Abstract

Introduction

Hypertension, one of the most prevalent non-communicable diseases in West Africa, can be well managed with good primary care. This scoping review will explore what is documented in the literature about factors that influence primary care access, utilization, and quality of management for patients living with hypertension in West Africa.

Methods and analysis

The scoping review will employ the approach described by Arksey and O'Malley (2005). The approach has five stages: (1) formulating the research questions, (2) identifying relevant studies, (3) selecting eligible studies, (4) charting the data and (5) collating, summarizing, and reporting the results. This review will employ the PRISMA extension for scoping reviews to report the results. PubMed, Embase, Scopus, Cairn Info, and Google Scholar will be searched for publications from 1st January 2000 to 31st December 2023. Studies reported English, French or Portuguese will be considered for inclusion. Research articles, systematic reviews, observational studies, and reports that include information on the relevant factors that influence primary care management of hypertension in West Africa will be eligible for inclusion. Study participants should be adults (aged 18 years or older). Clinical case series/case reports, short communications, books, grey literature and conference proceedings will be excluded. Papers on gestational hypertension and pre-eclampsia will be excluded.

Ethics and dissemination

This review does not require ethics approval. Our dissemination strategy includes peer-reviewed publications, policy briefs, presentations at conferences, dissemination to stakeholders, and intervention co-production forums.

Strengths and limitations of this study

For peer review only - http://bmjopen.bmj.com/site/about/guidelines.xhtml

- This scoping review will be useful for identifying areas of needed intervention and research in the primary care management of hypertension in West Africa
- This study will also help identify strengths, weaknesses, and areas of challenges in the access, utilization, and management of hypertension in primary care facilities.
- There is a possibility of bias because the review will only include studies in the official languages used in West Africa (English, French, and Portuguese).

INTRODUCTION

Background

Cardiovascular disease accounts for 17.9 million deaths every year,(1) most of which are in Low-and Middle-income countries(LMICs). In 2016 for example, 78% of all non communicable disease (NCD) deaths, and 85% of premature adult NCD deaths, were in LMICs.(2) An estimated 74.7 million individuals in sub Saharan Africa are hypertensive and this number is projected to increase to 125.5 million by 2025.(3)

Hypertension, also known as raised blood pressure, is diagnosed when blood pressure is measured on two different days and the systolic blood pressure readings on both days is greater or equal to 140 mmHg and/or the diastolic blood pressure readings on both days is greater or equal to 90 mmHg.(4) Hypertension is a precursor for various diseases such as ischemic and hemorrhagic stroke, chronic kidney disease, and coronary artery disease.(5) In 2015, 27% of the population in the African region had high blood pressure.(2) Hypertension is common in West Africa.(6) A study on hypertension prevalence, awareness, treatment and control done in seven communities in east and west Africa comprising 3547 participants, showed that a quarter of participants had hypertension, about 40% were unaware, half of those aware were treated, and half of those treated had controlled blood pressure.(7) Another study on the rural-urban difference in the prevalence of hypertension in West Africa showed prevalence of hypertension was 32.6% in males, and 30.0% in females with a lower odd of hypertension in rural compared to urban dwellers.(8)

Primary care is defined by the World Health Organization (WHO) as ‘a model of care that supports first-contact, accessible, continuous, comprehensive and coordinated person-focused care’.(9) It is the most basic form of care available to patients at the base of the health care pyramid. It should be accessible to all citizens and be able to provide primary and secondary

For peer review only - http://bmjopen.bmj.com/site/about/guidelines.xhtml

prevention for common diseases in the community. Primary care is very important because it has been shown to meet the needs of majority of people throughout their lives.(9) With the increasing emergence of NCDs as a health problem, there is the need to strengthen primary care in their management in the West African sub-region. This scoping review explores access, utilization, and quality of primary care for hypertension management to contribute evidence for strengthening primary care for one of the most common NCDs in West Africa.

Health care access is “concerned with helping people to command appropriate health care resources in order to preserve or improve their health”. Access requires adequate service quality and availability or supply, as well as removal of financial, organisational, social and cultural barriers that can limit service use(10) even when it is in adequate supply.(11) Access to healthcare for hypertension in West Africa is often quite low for multiple reasons such as cost, lack of knowledge, essential equipment, tools and supplies as well as medicines.(12,13) People use health care for many reasons including preventing and curing health problems, promoting maintenance of health and well-being, or obtaining information about their health status and prognosis. Access and utilization or use of healthcare go hand in hand, as good access to healthcare improves utilization. Quality of care according to the WHO is the degree to which health services for individuals and populations increase the likelihood of desired health outcomes.(14) Quality primary health care should be timely, equitable, integrated and efficient. These 3 components- access, utilization and quality of care have intersecting features that come together to ensure that patients receive the care they need. Good access to and utilization of adequate quality primary care for people living with hypertension can potentially reduce complications and avoidable hospitalization and mortality.(15,16) Weak and under-resourced health systems and the marginalization of NCD care in low- and middle-income countries (LMIC) make it harder to ensure prevention, early diagnosis, access to and utilization of adequate quality primary care and treatment of patients with hypertension.

The Economic Community of West Africa states (ECOWAS) sub-region is made up of 15 LMICs who share similar social, economic, and political environments. The countries in the ECOWAS are Benin, Burkina Faso, Cabo Verde, Cote d'Ivoire, The Gambia, Ghana, Guinea, Guinea-Bissau, Liberia, Mali, Niger, Nigeria, Senegal, Sierra Leone, and Togo.(17) A study of 192,441 hypertensive patients in 44 LMIC, including countries in West Africa, to determine the cascade of hypertension care – and its variation between countries and population groups showed that only 18%–29.9% of participants received treatment for hypertension, with 5%–10.3% achieving control of their hypertension.(18) Countries in the

For peer review only - http://bmjopen.bmj.com/site/about/guidelines.xhtml

Caribbean and Latin America did better than countries in Sub Saharan Africa. Hypertension control encompasses not just medications but also preventive practices like lifestyle and dietary changes.(1) To achieve SDG goal 3.4 i.e., to reduce by one-third premature mortality from NCDs through prevention and treatment, there is the need to explore the barriers at the primary care level in West Africa.

Contextual factors are a range of factors that make up the wider setting within which the individual and the health system exist. They include personal, social, cultural, economic, and political factors that have differing impacts across population groups.(19) In this study, we analyze context using the four categories in Leichter's framework.(20) These are Situational factors such as wars, famine, epidemics, political instability; Structural factors such as economic structure, political system, technological change, degree of urbanization, structure of the labor markets and demographic structure; Cultural factors such as the level of literacy, and values on issues such as religion, gender, participation and corruption; and Environmental or international/exogenous factors external to the political system such as the role of transnational companies and international agreements and events. These contextual factors influence access to care and individual's decision to seek and maintain treatment for hypertension and are therefore relevant in exploring the barriers to hypertension care at primary care level in West Africa.

Individual, community, primary health care facility and health system factors also influence access, quality and utilization. Some individual/patient factors like medication non-adherence, poor lifestyle and dietary choices, lack of awareness and funds to purchase medications leads to poor management of high blood pressure.(21–23) Health system challenges such as poor referral systems, inadequate tools and equipment, insufficient funding and lack of sufficient health personnel poses challenges in the access to care and quality of management of hypertension at primary care facilities.(22,24,25)

Rationale

There is an urgent need to explore the available data on the barriers and enablers to the control of hypertension in West Africa especially as part of primary care. Although a similar scoping review was done on the reasons for poor blood pressure control in eastern Sub-Saharan Africa(SSA) focusing on patient, professional, primary healthcare system, and public health policy challenges,(26) there is no similar study for Western SSA. Also, the study in eastern SSA did not explore contextual factors that influence access, utilization, and quality

For peer review only - http://bmjopen.bmj.com/site/about/guidelines.xhtml

For peer review only

of primary care for hypertension. For the patients as well as health practitioners, such a review in West Africa will serve to inform understanding of the challenges in the management of hypertension in primary care facilities. The findings will also be key in informing co-production of interventions as well as identifying areas of needed research in the primary care management of hypertension in West Africa.

Objectives

This review therefore aims to synthesize what is documented in the literature about the factors that hinder and or enable primary care for hypertension at individual, contextual, health facility and health system levels in West Africa and how and why they work. This includes access, utilization as well as quality of available services.

The specific objectives of this study are to:

1. Describe the factors documented in the literature that hinder and/or enable management of hypertension at primary care level in West Africa (including access, utilization, and quality of care); and how and why they work.

    1.1. Describe contextual factors (national and global, socio-cultural, socio-economic, political) and analyze how and why they work.

    1.2. Describe individual/patient factors and analyze how and why they work.

    1.3. Describe health facility and health system factors and analyze how and why they work.

2. To identify relevant gaps in the existing literature and areas of research interest, in the primary care of hypertension in West Africa.

Conceptual framework

A conceptual framework of factors that influence the primary care access, utilization and quality of care for people living with hypertension in west Africa has been developed to help structure our review as illustrated in Figure 1. This framework was adapted from a study on facilitators and barriers to NCD prevention in Pakistan(27) and modified with information from additional studies.(20,28,29)

METHODS AND ANALYSIS

For peer review only - http://bmjopen.bmj.com/site/about/guidelines.xhtml

Study design

A scoping review methodology as proposed by Arksey and O'Malley(2005)(30) will be used due to the broad nature of the research question and the uncertainty of the nature and quality of the evidence available to answer the questions. The approach consists of five stages: (1) formulating the research questions, (2) identifying relevant studies, (3) selecting eligible studies, (4) charting the data and (5) collating, summarizing, and reporting the results. The scoping review also draws inspiration from methodology developed by the Joanna Briggs Institute.(31) Scoping reviews are useful in presenting a broad overview of the data available on a topic and allows one to identify gaps in knowledge and research.(32) They also help to determine if a systematic review would be relevant in the area of research. The review will apply the PRISMA extension for scoping reviews to present the results.(33)

The research question was obtained through discussion with key health system stakeholders as well as researchers in the STOP NCD project, which started implementation in October 2022. The protocol was not registered on Prospero as Prospero currently does not allow for registration of scoping review protocols.

Identifying the research question

We used an iterative process, as recommended by Arksey and O'Malley, to develop the research question through consultations with the research team and key health system stakeholders. The overall main research question is defined as:

What are the factors (at the individual, community, health system and context level) that influence access, utilization and primary care management of people living with hypertension in West Africa and how and why do they work?

Identifying relevant studies

Searches will be conducted using PubMed, Embase, Scopus, Cairn Info (primarily for French papers) and google scholar databases for the period of 1 January 2000 to 31 December 2023 with the help of a librarian. This time frame was selected because there has been a surge in the burden of NCDs in sub-Saharan Africa over the past two decades, driven by increasing incidence of cardiovascular risk factors such as unhealthy diets, reduced physical activity, hypertension, obesity, diabetes, dyslipidemia, and air pollution.(34) The key concepts from our research question and alternative search terms (see Appendix 1) will be combined using

For peer review only - http://bmjopen.bmj.com/site/about/guidelines.xhtml

the Boolean terms AND, NOT and OR. Excel power query will be used to generate all combinations of the key search terms to perform an exhaustive search. The search strategies we are using for the various databases is shown in **Error! Reference source not found.** 2. The search strategy will be adapted as appropriate for each of the search databases. Only published literature will be included in the review. Other literature may be found using the software 'research rabbit' by inputting relevant papers. Some literature may also be found by searching the reference list and citations of key articles used in the review. Relevant websites such as the WHO website will be accessed for relevant literature and included in the study. The search results will be exported into Rayyan for removal of duplicates and abstracts screening.

Study selection

The studies found will be screened against the pre-specified eligibility criteria and study selection will be done by two independent researcher pairs. The studies will first be examined with their titles and abstract, and studies that are obviously irrelevant will be removed. Relevant studies will be retrieved and examined fully to determine eligibility. Any disagreements will be resolved through discussion and where necessary, involvement of a third independent researcher. There will be an initial pilot selection of a few papers to test the search eligibility criteria prior to the start of the main selection. This will be done by two independent reviewers to ensure reliability. The final results of the search will be reported in full in the final report and presented in a PRISMA flow diagram.(35)(Appendix 3) The reason for excluding any studies will be documented and included in our final report. Additionally, a description of the PCCS framework which stands for population, concept, context, and study elements is outlined (

Table 1) to guide the screening and identification of relevant studies.

Table 1. Population, concept, context, and study elements

Population	Concept	Context	Study
Adults 18 years or older with systemic hypertension	Hindering and enabling factors that influence	Macro (National and global level factors)	Qualitative, quantitative, meta-analysis, social

For peer review only - http://bmjopen.bmj.com/site/about/guidelines.xhtml

in West Africa	first level of care for hypertension	Meso (Health System factors including Primary care facilities Micro (Community factors)	science case studies and case series

Inclusion criteria

Relevant papers written between 1 January 2000 to 31 December 2023 with participants over the age of 18 years will be included.

Articles, systematic reviews, observational studies, and reports that include information on the relevant factors that influence primary care management of hypertension in West Africa will be included in the paper.

Papers written in English, French or Portuguese (Guinea-Bissau and Cape Verde) will be included.

Exclusion criteria

Clinical case series/case reports, short communications, books, grey literature, and conference proceedings will be excluded.

Papers on gestational hypertension and pre-eclampsia will be excluded.

Data charting

The data that will be charted will be entered onto a 'data charting form' using the database program Excel. The data charting form will include the name of the author, study type, year of publication, study design, study population, sample size, study objectives, type of evidence source (i.e., primary study, review, website etc.) key findings, comments, and limitations. An illustration is shown in Appendix 4. Two independent reviewer pairs will test the data charting form on a few studies to ensure validity. Any disagreement will be settled by consensus or through the involvement of a third independent reviewer.

For peer review only - http://bmjopen.bmj.com/site/about/guidelines.xhtml

For peer review only

Collating, summarizing, and reporting the results

The available evidence on the factors that influence primary care for hypertensive patients will be described and summarized under themes with the following headings: individual, contextual, health systems/health facility and gaps in the research. Individual factors include lifestyle, dietary habits and risk factors that impede or encourage management of hypertension. These factors include patient's adherence to medications and recommended lifestyle changes and barriers to achieving hypertension control. Contextual factors will be explored at macro (national and global levels), meso (sub-national including health system and facility levels), and micro (community and household levels). At the macro level this is summarized as the socio-cultural, socio-economic, and political environment that hinder/enable primary care management of hypertension management using Leichter's framework. At the meso level, the health system and health facility factors are the issues that enable/prevent primary care management for patients living with hypertension. The data collected from the included studies will be tabulated and summarized narratively.

Patient and public involvement

None.

ETHICS AND DISSEMINATION

This review does not require ethics approval. Our dissemination strategy includes peer-reviewed publication, policy brief, presentation at conferences, dissemination to relevant stakeholders, and intervention co-production forums.

For peer review only

Acknowledgements

We thank colleagues on the Stop-NCD context analysis team for their comments and feedback on the earlier drafts of this protocol.

Funding

This work received funding from NIHR (National Institute for health research) global Health Research centers project: Research and Institutional capacity Strengthening in NCDs. Grant number NIHR203246 – Global Health Research Centre on Strengthening of Capacity for NCD control in West Africa (Stop-NCD).

Competing interests

None declared.

Contributors

KAA drafted the research question, study design and methods and final editing, drafting and submission of the protocol. EPKB, VR, IA, EKA, AD, TM, PP, MY and EA aided in developing the research question and study methods, contributed meaningfully to the drafting and editing, and approved the final manuscript.

References

1. Non communicable diseases [Internet]. [cited 2023 Mar 15]. Available from: https://www.who.int/news-room/fact-sheets/detail/noncommunicable-diseases

2. World Health Organization. Noncommunicable diseases country profiles 2018 [Internet]. Geneva: World Health Organization; 2018 [cited 2023 Mar 14]. 223 p. Available from: https://apps.who.int/iris/handle/10665/274512

3. Ogah OS, Rayner BL. Recent advances in hypertension in sub-Saharan Africa. Heart. 2013 Oct;99(19):1390–7.

4. Hypertension [Internet]. [cited 2023 Mar 14]. Available from: https://www.who.int/news-room/fact-sheets/detail/hypertension

5. A GLOBAL BRIEF ON HYPERTENSION WHO.pdf.

For peer review only

6. Dalal S, Beunza JJ, Volmink J, Adebamowo C, Bajunirwe F, Njelekela M, et al. Non-communicable diseases in sub-Saharan Africa: what we know now. Int J Epidemiol. 2011 Aug;40(4):885–901.

7. Okello S, Muhihi A, Mohamed SF, Ameh S, Ochimana C, Oluwasanu AO, et al. Hypertension prevalence, awareness, treatment, and control and predicted 10-year CVD risk: a cross-sectional study of seven communities in East and West Africa (SevenCEWA). BMC Public Health. 2020 Nov 13;20(1):1706.

8. Sani RN, Connelly PJ, Toft M, Rowa-Dewar N, Delles C, Gasevic D, et al. Rural-urban difference in the prevalence of hypertension in West Africa: a systematic review and meta-analysis. J Hum Hypertens. 2022 Apr 16;1–13.

9. Primary care [Internet]. [cited 2023 Apr 27]. Available from: https://www.who.int/teams/integrated-health-services/clinical-services-and-systems/primary-care

10. Carrasquillo O. Health Care Utilization. In: Gellman MD, Turner JR, editors. Encyclopedia of Behavioral Medicine [Internet]. New York, NY: Springer; 2013 [cited 2023 Oct 7]. p. 909–10. Available from: https://doi.org/10.1007/978-1-4419-1005-9_885

11. Gulliford M, Figueroa-Munoz J, Morgan M, Hughes D, Gibson B, Beech R, et al. What does "access to health care" mean? J Health Serv Res Policy. 2002 Jul 1;7(3):186–8.

12. Odland ML, Bockarie T, Wurie H, Ansumana R, Lamin J, Nugent R, et al. Prevalence and access to care for cardiovascular risk factors in older people in Sierra Leone: a cross-sectional survey. BMJ Open. 2020 Sep 9;10(9):e038520.

13. Adeke AS, Umeokonkwo CD, Balogun MS, Odili AN. Essential medicines and technology for hypertension in primary healthcare facilities in Ebonyi State, Nigeria. PLoS One. 2022;17(2):e0263394.

14. Quality of care [Internet]. [cited 2023 Oct 7]. Available from: https://www.who.int/health-topics/quality-of-care

15. Walker RL, Chen G, McAlister FA, Campbell NRC, Hemmelgarn BR, Dixon E, et al. Hospitalization for Uncomplicated Hypertension: An Ambulatory Care Sensitive Condition. Canadian Journal of Cardiology. 2013 Nov 1;29(11):1462–9.

16. Kuecker CM, Kashyap A, Seckel E. Implementation of a Protocol to Manage Patients at Risk for Hospitalization Due to an Ambulatory Care Sensitive Condition. Fed Pract. 2020 Aug;37(8):380–3.

17. United States Trade Representative [Internet]. [cited 2023 Jun 12]. Economic Community of West African States (ECOWAS). Available from: http://ustr.gov/countries-regions/africa/regional-economic-communities-rec/economic-community-west-african-states

18. Geldsetzer P, Manne-Goehler J, Marcus ME, Ebert C, Zhumadilov Z, Wesseh CS, et al. The state of hypertension care in 44 low-income and middle-income countries: a cross-sectional study of nationally representative individual-level data from 1·1 million adults. Lancet. 2019 Aug 24;394(10199):652–62.

For peer review only - http://bmjopen.bmj.com/site/about/guidelines.xhtml

19. Glossary [Internet]. [cited 2023 Apr 26]. Available from: https://www.australiancurriculum.edu.au/f-10-curriculum/health-and-physical-education/glossary/

20. Leichter HM. A Comparative Approach to Policy Analysis: Health Care Policy in Four Nations. CUP Archive; 1979. 340 p.

21. Atinga RA, Yarney L, Gavu NM. Factors influencing long-term medication non-adherence among diabetes and hypertensive patients in Ghana: A qualitative investigation. PLOS ONE. 2018 Mar 28;13(3):e0193995.

22. Nyaaba GN, Masana L, de-Graft Aikins A, Beune E, Agyemang C. Factors hindering hypertension control: perspectives of front-line health professionals in rural Ghana. Public Health. 2020 Apr;181:16–23.

23. Buabeng KO. Unaffordable drug prices: the major cause of non-compliance with hypertension medication in Ghana. 2004;

24. Vialle-Valentin CE, Serumaga B, Wagner AK, Ross-Degnan D. Evidence on access to medicines for chronic diseases from household surveys in five low- and middle-income countries. Health Policy Plan. 2015 Oct;30(8):1044–52.

25. Iwelunmor J, Blackstone S, Gyamfi J, Airhihenbuwa C, Plange-Rhule J, Tayo B, et al. A Concept Mapping Study of Physicians' Perceptions of Factors Influencing Management and Control of Hypertension in Sub-Saharan Africa. International Journal of Hypertension. 2015 Oct 13;2015:e412804.

26. Link to external site this link will open in a new window. Reasons for poor blood pressure control in Eastern Sub-Saharan Africa: looking into 4P's (primary care, professional, patient, and public health policy) for improving blood pressure control: a scoping review. 2021;1–15.

27. Facilitators and barriers to NCD prevention in Pakistanis–invincibility or inevitability: a qualitative research study | BMC Research Notes | Full Text [Internet]. [cited 2023 May 5]. Available from: https://bmcresnotes.biomedcentral.com/articles/10.1186/s13104-016-2087-2

28. Koduah A, Nonvignon J, Colson A, Kurdi A, Morton A, van der Meer R, et al. Health systems, population and patient challenges for achieving universal health coverage for hypertension in Ghana. Health Policy and Planning. 2021 Nov 1;36(9):1451–8.

29. Sorato MM, Davari M, Kebriaeezadeh A, Sarrafzadegan N, Shibru T, Fatemi B. Reasons for poor blood pressure control in Eastern Sub-Saharan Africa: looking into 4P's (primary care, professional, patient, and public health policy) for improving blood pressure control: a scoping review. BMC Cardiovascular Disorders. 2021 Mar 4;21(1):123.

30. Arksey H, O'Malley L. Scoping Studies: Towards a Methodological Framework. International Journal of Social Research Methodology: Theory & Practice. 2005;8:19–32.

For peer review only - http://bmjopen.bmj.com/site/about/guidelines.xhtml

31. Chapter 11: Scoping reviews. In: JBI Manual for Evidence Synthesis [Internet]. JBI; 2020 [cited 2023 Jun 7]. Available from: https://jbi-global-wiki.refined.site/space/MANUAL/4687342/Chapter+11%3A+Scoping+reviews

32. Tricco AC, Lillie E, Zarin W, O'Brien K, Colquhoun H, Kastner M, et al. A scoping review on the conduct and reporting of scoping reviews. BMC Medical Research Methodology. 2016 Feb 9;16(1):15.

33. Tricco AC, Lillie E, Zarin W, O'Brien KK, Colquhoun H, Levac D, et al. PRISMA Extension for Scoping Reviews (PRISMA-ScR): Checklist and Explanation. Ann Intern Med. 2018 Oct 2;169(7):467–73.

34. Bigna JJ, Noubiap JJ. The rising burden of non-communicable diseases in sub-Saharan Africa. The Lancet Global Health. 2019 Oct;7(10):e1295–6.

35. Page MJ, McKenzie JE, Bossuyt PM, Boutron I, Hoffmann TC, Mulrow CD, et al. The PRISMA 2020 statement: an updated guideline for reporting systematic reviews. BMJ. 2021 Mar 29;372:n71.

FIGURE TITLE

Figure 1. Conceptual framework on the factors that influence primary care access, utilization and quality for people living with hypertension

For peer review only

For peer review only - http://bmjopen.bmj.com/site/about/guidelines.xhtml

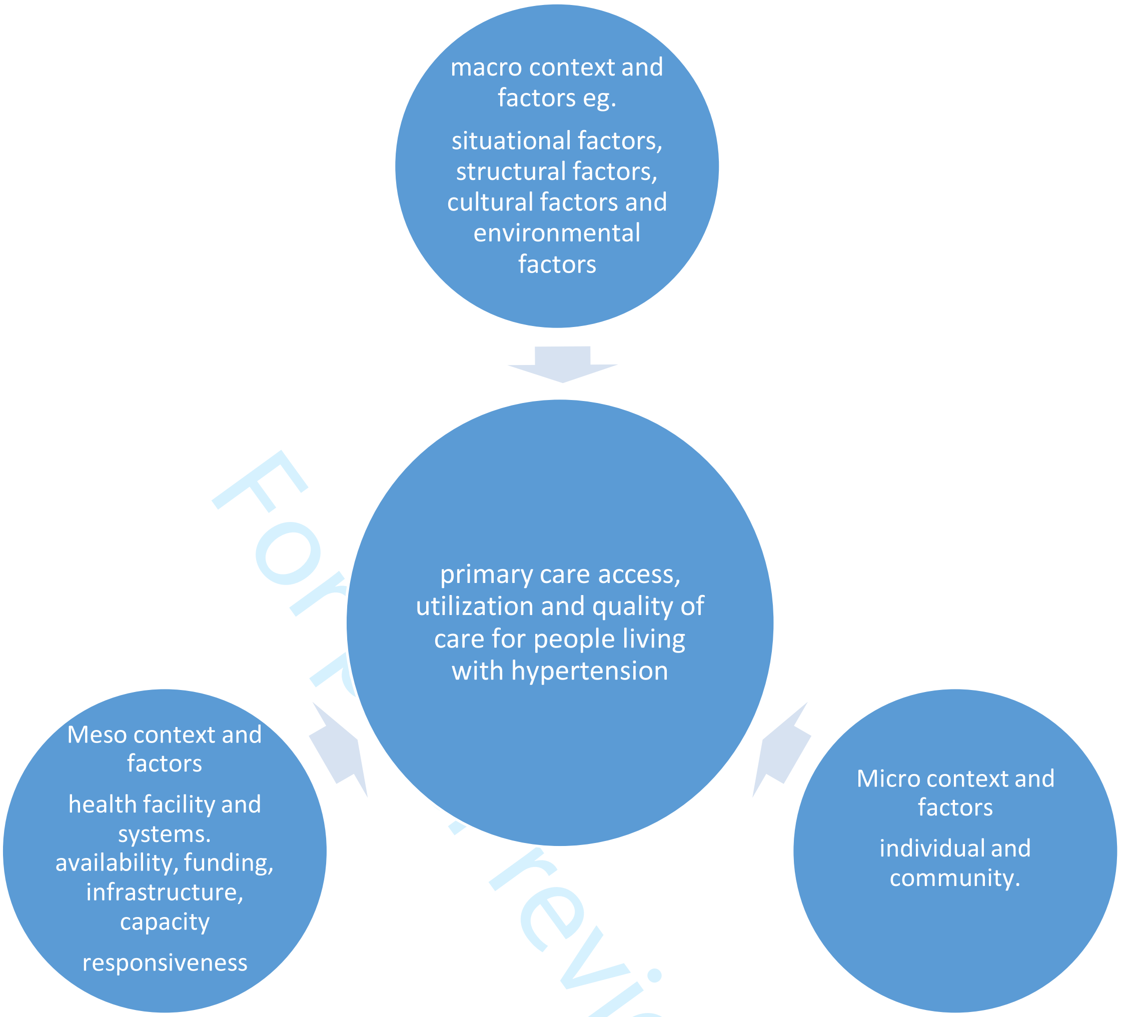

Figure 1-conceptual framework on the factors that influence primary care access, utilization and quality for people living with hypertension

Appendices

Appendix 1- Key concepts and alternative terms.

Search concepts	Alternatives terms
1. Factors	Socio-cultural, socio-economic, social, societal, health systems, individual, patient, community, political
2. Influence	Hinder, enable, promote, threaten, impact, control, motivate, affect,
3.Primary care	Screening, medications, counselling, patient education, prescription, outpatient visits, lifestyle, access, utilization, quality of care
4. Hypertension	High blood pressure, raised blood pressure, increased blood pressure, hypertensive disease, hypertension diagnosis, hypertension treatment, hypertension control, hypertension cascade, NCD management
5. West Africa	Ghana, Niger, Burkina Faso, Benin, Cape Verde, Côte D'Ivoire, Gambia, Guinea, Guinea-Bissau, Liberia, Mali, Mauritania, Nigeria, Senegal, Sierra Leone, Togo

For peer review only

Appendix 2- search strategy for various databases

Search terms for google scholar	comments
(hinder OR enable OR promote OR influence OR affect) AND Hypertension AND "quality of care" AND "primary care" AND "West Africa" hypertension -pregnancy, -gestational	Must include – hypertension<br><br>Exclude pregnancy, gestational
(hinder OR enable OR promote OR influence OR affect) AND Hypertension AND "quality of care" AND "primary care" AND "West Africa" "hypertension" -pregnancy, -gestational	Must include – hypertension<br><br>Exclude pregnancy, gestational
(Access OR utilization) AND "high blood pressure" AND "quality of care" AND "primary care" AND "West Africa" "hypertension" -pregnancy, -gestation,	Must include – hypertension<br><br>Exclude pregnancy, gestational
(hinder OR enable OR promote OR influence OR affect) AND (access OR utilization) AND "High blood pressure" AND "quality of care" AND "primary care" AND "West Africa" hypertension -pregnancy, -gestational	Must include – hypertension<br><br>Exclude pregnancy, gestational
(Socio-cultural OR socioeconomic OR individual OR community OR "health system" OR "health financing") AND (Access OR utilization) AND "high blood pressure" AND "quality of care" AND "primary care" AND "West Africa" hypertension -pregnancy, -gestational	Must include – hypertension<br><br>Exclude pregnancy, gestational
Search query for PubMed / Embase	
((((hinder OR enable OR promote OR influence OR affect[MeSH Terms]) AND ((Hypertension[MeSH Terms]) OR ((((((((("high blood pressure") OR ("raised blood pressure")) OR ("increased blood pressure")) OR ("hypertensive disease")) OR ("hypertension diagnosis")) OR ("hypertension treatment")) OR ("hypertension control")) OR ("hypertension cascade")) OR ("NCD management")))) AND ((primary care[MeSH Terms]) OR ((((((((((screening) OR (medication*)) OR (counselling)) OR ("patient education")) OR (prescription)) OR ("outpatient visit*")) OR (lifestyle)) OR (access)) OR (utilisation)) OR ("quality of care")))) AND ((West Africa[MeSH Terms]) OR ((((((((((((((((Togo) OR ("Sierra Leone")) OR (Senegal)) OR (Nigeria)) OR (Mauritania)) OR (Mali)) OR (Liberia)) OR ("Guinea-Bissau")) OR (Guinea)) OR (Gambia)) OR ("Côte D'Ivoire")) OR ("Cape Verde")) OR (Benin)) OR ("Burkina Faso")) OR (Niger)) OR (Ghana)))) OR (((((hinder OR enable OR promote OR influence OR affect[MeSH Terms]) AND ((Hypertension[MeSH Terms]) OR ((((((((("high blood pressure") OR ("raised blood pressure")) OR ("increased blood pressure")) OR ("hypertensive disease")) OR ("hypertension diagnosis")) OR ("hypertension treatment")) OR ("hypertension control")) OR ("hypertension cascade")) OR ("NCD management")))) AND ((primary care[MeSH Terms]) OR ((((((((((screening) OR (medication*)) OR (counselling)) OR ("patient education")) OR (prescription)) OR ("outpatient visit*")) OR (lifestyle)) OR (access)) OR (utilisation)) OR ("quality of care")))) AND (((quality of health care[MeSH Terms]) OR (health care quality[MeSH Terms])) OR (health care quality, access, and evaluation[MeSH Terms]))) AND ((West Africa[MeSH Terms]) OR ((((((((((((((((Togo) OR ("Sierra Leone")) OR (Senegal)) OR (Nigeria)) OR (Mauritania)) OR (Mali)) OR (Liberia)) OR ("Guinea-Bissau")) OR	

For peer review only

For peer review only - http://bmjopen.bmj.com/site/about/guidelines.xhtml

(Guinea)) OR (Gambia)) OR ("Côte D'Ivoire")) OR ("Cape Verde")) OR (Benin)) OR ("Burkina Faso")) OR (Niger)) OR (Ghana))))	
((((Socio-cultural OR socioeconomic OR individual OR community OR "health system" OR "health Insurance") AND ((Hypertension[MeSH Terms]) OR (((((((("high blood pressure") OR ("raised blood pressure")) OR ("increased blood pressure")) OR ("hypertensive disease")) OR ("hypertension diagnosis")) OR ("hypertension treatment")) OR ("hypertension control")) OR ("hypertension cascade")) OR ("NCD management")))) AND ((primary care[MeSH Terms]) OR (((((((((screening) OR (medication*)) OR (counselling)) OR ("patient education")) OR (prescription)) OR ("outpatient visit*")) OR (lifestyle)) OR (access)) OR (utilisation)) OR ("quality of care")))) AND ((West Africa[MeSH Terms]) OR ((((((((((((((Togo) OR ("Sierra Leone")) OR (Senegal)) OR (Nigeria)) OR (Mauritania)) OR (Mali)) OR (Liberia)) OR ("Guinea-Bissau")) OR (Guinea)) OR (Gambia)) OR ("Côte D'Ivoire")) OR ("Cape Verde")) OR (Benin)) OR ("Burkina Faso")) OR (Niger)) OR (Ghana)))) OR (((((Socio-cultural OR socioeconomic OR individual OR community OR "health system" OR "health Insurance") AND ((Hypertension[MeSH Terms]) OR (((((((("high blood pressure") OR ("raised blood pressure")) OR ("increased blood pressure")) OR ("hypertensive disease")) OR ("hypertension diagnosis")) OR ("hypertension treatment")) OR ("hypertension control")) OR ("hypertension cascade")) OR ("NCD management")))) AND ((primary care[MeSH Terms]) OR (((((((((screening) OR (medication*)) OR (counselling)) OR ("patient education")) OR (prescription)) OR ("outpatient visit*")) OR (lifestyle)) OR (access)) OR (utilisation)) OR ("quality of care")))) AND (((quality of health care[MeSH Terms]) OR (health care quality[MeSH Terms])) OR (health care quality, access, and evaluation[MeSH Terms]))) AND ((West Africa[MeSH Terms]) OR ((((((((((((((Togo) OR ("Sierra Leone")) OR (Senegal)) OR (Nigeria)) OR (Mauritania)) OR (Mali)) OR (Liberia)) OR ("Guinea-Bissau")) OR (Guinea)) OR (Gambia)) OR ("Côte D'Ivoire")) OR ("Cape Verde")) OR (Benin)) OR ("Burkina Faso")) OR (Niger)) OR (Ghana))))	
Search query for Scopus	
hypertension AND (hinder OR enable OR promote OR influe nee OR affect) AND managem ent AND ("primary care" OR "p rimary healthcare") AND "West Africa" OR ghana OR niger 0R "Burkina Faso" OR benin 0R "Cape Verde" OR "Cote D/3zap os;lvoire" OR gambia OR guin ea OR "Guinea-Bissau" OR libe ria OR mali OR mauritania 0 R nigeria OR senegal OR "Sier ra Leone" OR toga AND (LIMIT-TO (AFFILCOUNTRY, "Nigeri a") OR LIMIT-TO (AFFILCOUN TRY, "Ghana") OR LIMIT-T0 (AFFILCOUNTRY, "Benin") OR LIMIT-TO (AFFILCOUNTR Y, "Gambia") OR LIMIT-TO(AFFILCOUNTRY, "Burkina Faso") OR LIMIT-TO (AFFILCOUNTRY, "Senegal") OR LIMIT-T0 (AFFILCOUNTRY, "Liberia") OR LIMIT-TO (AFFILCOUNTRY, "Sierra Leone") OR LIMIT-T0 (AFFILCOUNTRY, "Cote d8{a pos;lvoire") OR LIMIT-TO (AFFI LCOUNTRY, "Togo") OR LIMI T-TO (AFFILCOUNTRY, "Mali") OR LIMIT-TO (AFFILCOUNTR Y, "Niger") OR LIMIT-TO (AFFILCOUNTRY, "Cape Verde") 0 R LIMIT-TO (AFFILCOUNTRY, "Guinea") OR LIMIT-TO(AFFILCOUNTRY, "Guinea-Bissau") OR LIMIT-TO (AFFILCOUNTRY, "Mauritania")) AND (LIMIT-TO (PUBYEAR, 2023) OR LI MIT-TO (PUBYEAR, 2022) OR LIMIT-TO (PUBYEAR, 2021) 0 R LIMIT-TO (PUBYEAR, 2020) OR LIMIT-TO (PUBYEAR, 201 9) OR LIMIT-TO (PUBYEAR, 2018) OR LIMIT-TO (PUBYEA R, 2017) OR LIMIT-TO (PUBY EAR, 2016) OR LIMIT-TO (PU BYEAR, 2015) OR LIMIT-T0 (PUBYEAR, 2014) OR LIMIT-TO (PUBYEAR,	

For peer review only

For peer review only - http://bmjopen.bmj.com/site/about/guidelines.xhtml

2013) OR LI MIT-TO (PUBYEAR, 2012) OR LIMIT-TO (PUBYEAR, 2011) 0 R LIMIT-TO (PUBYEAR, 2010) OR LIMIT-TO (PUBYEAR, 200 9) OR LIMIT-TO (PUBYEAR, 2008) OR LIMIT-TO (PUBYEA R, 2007) OR LIMIT-TO (PUBY EAR, 2006) OR LIMIT-TO (PU BYEAR, 2005) OR LIMIT-T 0 (PUBYEAR, 2004) OR LIMI T-TO (PUBYEAR, 2003) OR LI MIT-TO (PUBYEAR, 2002) OR LIMIT-TO (PUBYEAR, 2001) 0 R LIMIT-TO (PUBYEAR, 200 0))	
hypertension AND (hinder OR enable OR promote OR influenee OR affect) AND ("quality of care") AND ("primary care" OR "primary healthcare") AND "West Africa" OR ghana OR nig er OR "Burkina Faso" OR beni n OR "Cape Verde" OR "Cote D'lvoire" OR gambia OR g uinea OR "Guinea-Bissau" OR liberia OR mali OR mauritania OR nigeria OR senegal OR "Sierra Leone" OR toga AND (LIMIT-TO (AFFILCOUNTRY,"Nigeria") OR LIMIT-TO (AFFIL COUNTRY, "Ghana") OR LIMI T-TO (AFFILCOUNTRY, "Beni n") OR LIMIT-TO (AFFILCOUN TRY, "Gambia") OR LIMIT-T0 (AFFILCOUNTRY, "Burkina F aso") OR LIMIT-TO (AFFILCOU NTRY, "Senegal") OR LIMIT-T0 (AFFILCOUNTRY, "Liberia") OR LIMIT-TO (AFFILCOUNTRY, "Sierra Leone") OR LIMIT-T0 (AFFILCOUNTRY, "Cote dll{apos;lvoire") OR LIMIT-TO (AFFI LCOUNTRY, "Togo") OR LIMI T-TO (AFFILCOUNTRY, "Mali") OR LIMIT-TO (AFFILCOUNTR Y, "Niger") OR LIMIT-TO (AFFILCOUNTRY, "Cape Verde") 0 R LIMIT-TO (AFFILCOUNTRY, "Guinea") OR LIMIT-TO(AFFILCOUNTRY, "Guinea-Bissau") OR LIMIT-TO (AFFILCOUNTRY, "Mauritania")) AND (LIMIT-TO (PUBYEAR, 2023) OR LI MIT-TO (PUBYEAR, 2022) OR LIMIT-TO (PUBYEAR, 2021) 0 R LIMIT-TO (PUBYEAR, 2020) OR LIMIT-TO (PUBYEAR, 201 9) OR LIMIT-TO (PUBYEAR, 2018) OR LIMIT-TO (PUBYEA R, 2017) OR LIMIT-TO (PUBY EAR, 2016) OR LIMIT-TO (PU BYEAR, 2015) OR LIMIT-T0 (PUBYEAR, 2014) OR LIMIT-TO (PUBYEAR, 2013) OR LI MIT-TO (PUBYEAR, 2012) OR LIMIT-TO (PUBYEAR, 2011) 0 R LIMIT-TO (PUBYEAR, 2010) OR LIMIT-TO (PUBYEAR, 200 9) OR LIMIT-TO (PUBYEAR, 2008) OR LIMIT-TO (PUBYEA R, 2007) OR LIMIT-TO (PUBY EAR, 2006) OR LIMIT-TO (PU BYEAR, 2005) OR LIMIT-T0 (PUBYEAR, 2004) OR LIMI T-TO (PUBYEAR, 2003) OR LI MIT-TO (PUBYEAR, 2002) OR LIMIT-TO (PUBYEAR, 2001) 0 R LIMIT-TO (PUBYEAR, 200 0))	
hypertension AND (hinder OR enable OR promote OR influenee OR affect) AND (utilization OR access) AND ("primary care" OR "primary healthcare") AND "West Africa" OR ghana 0R niger OR "Burkina Faso" OR benin OR "Cape Verde" OR "C ate Dll{apos;lvoire" OR gambia OR guinea OR "Guinea-Bissa u" OR liberia OR mali OR ma uritania OR nigeria OR senega I OR "Sierra Leone" OR toga AND (LIMIT-TO(AFFILCOUNTR Y, "Nigeria") OR LIMIT-TO(A FFILCOUNTRY, "Ghana") OR LIMIT-TO (AFFILCOUNTRY, "B enin") OR LIMIT-TO (AFFILCO UNTRY, "Gambia") OR LIMIT- TO (AFFILCOUNTRY, "Burkina Faso") OR LIMIT-TO (AFFILCOUNTRY, "Senegal") OR LIMIT- TO (AFFILCOUNTRY, "Liberi a") OR LIMIT-TO (AFFILCOUNTRY, "Sierra Leone") OR LIMIT-TO (AFFILCOUNTRY, "Coted ll{apos;lvoire") OR LIMIT-TO (A FFILCOUNTRY, "Togo") OR LI MIT-TO (AFFILCOUNTRY, "Mal i") OR LIMIT-TO (AFFILCOUNT RY, "Niger") OR LIMIT-TO(AFFILCOUNTRY, "Cape Verde") OR LIMIT-TO (AFFILCOUNTR Y, "Guinea") OR LIMIT-TO(AFFILCOUNTRY, "Guinea-Bissa u") OR LIMIT-TO (AFFILCOUNTRY, "Mauritania")) AND (LI MIT-TO (PUBYEAR, 2023) OR LIMIT-TO (PUBYEAR, 2022) 0 R LIMIT-TO (PUBYEAR, 2021) OR LIMIT-TO (PUBYEAR, 202 0) OR LIMIT-TO (PUBYEAR, 2019) OR LIMIT-TO (PUBYEAR,2018) OR LIMIT-TO (PUBYEA R, 2017) OR LIMIT-TO (PUBY EAR, 2016) OR LIMIT-TO (PU BYEAR, 2015) OR LIMIT-T0 (PUBYEAR, 2014) OR LIMIT-TO (PUBYEAR, 2013) OR LI MIT-TO (PUBYEAR, 2012) OR LIMIT-TO (PUBYEAR, 2011) 0 R LIMIT-TO (PUBYEAR, 2010) OR LIMIT-TO (PUBYEAR, 200 9) OR LIMIT-TO (PUBYEAR, 2008) OR LIMIT-TO (PUBYEA R, 2007) OR LIMIT-TO (PUBY EAR, 2006) OR LIMIT-TO (PU BYEAR, 2005) OR LIMIT-T0 (PUBYEAR, 2004) OR LIMI T-TO (PUBYEAR, 2003) OR LI MIT-TO (PUBYEAR, 2002)	

For peer review only

OR LIMIT-TO (PUBYEAR, 2001) 0 R LIMIT-TO (PUBYEAR, 2000))	
hypertension AND (socio-cultural OR socioeconomic OR individual OR community OR "health system" OR "health financing") AND management AND ("primary care" OR "primary he althcare") AND "West Africa" 0 R ghana OR niger OR "Burkin a Faso" OR benin OR "Cape Verde" OR "Cote D'lvoire" OR gambia OR guinea OR "Guinea-Bissau" OR liberia OR mali OR mauritania OR nigeria OR senegal OR "Sierra Leone" OR toga AND (LIMIT-TO (AFFI LCOUNTRY, "Nigeria") OR LI MIT-TO (AFFILCOUNTRY, "Ghana") OR LIMIT-TO (AFFILCOUNTRY, "Benin") OR LIMIT-T0 (AFFILCOUNTRY, "Gambia") OR LIMIT-TO (AFFILCOUNTRY, "Burkina Faso") OR LIMI T-TO (AFFILCOUNTRY, "Senegal") OR LIMIT-TO (AFFILCOUN TRY, "Liberia") OR LIMIT-T0 (AFFILCOUNTRY, "Sierra Leo ne") OR LIMIT-TO(AFFILCOUNTRY, "Cote d'lvoire") 0 R LIMIT-TO(AFFILCOUNTRY, "Togo") OR LIMIT-TO (AFFILC OUNTRY, "Mali") OR LIMIT-T0 (AFFILCOUNTRY, "Niger") OR LIMIT-TO (AFFILCOUNTRY, "CapeVerde") OR LIMIT-T0 (AFFILCOUNTRY, "Guinea") OR LIMIT-TO (AFFILCOUNTRY, "Guinea-Bissau") OR LIMIT-TO (AFFILCOUNTRY, "Mauritania")) AND (LIMIT-TO(PUBYE AR, 2023) OR LIMIT-TO (PUB YEAR, 2022) OR LIMIT-TO (P UBYEAR, 2021) OR LIMIT-T0 (PUBYEAR, 2020) OR LIMI T-TO (PUBYEAR, 2019) OR LI MIT-TO (PUBYEAR, 2018) OR LIMIT-TO (PUBYEAR, 2017) 0 R LIMIT-TO (PUBYEAR, 2016) OR LIMIT-TO (PUBYEAR, 2015) OR LIMIT-TO (PUBYEAR, 2014) OR LIMIT-TO (PUBYEAR,2013) OR LIMIT-TO (PUBYEA R, 2012) OR LIMIT-TO (PUBY EAR, 2011) OR LIMIT-TO (PU BYEAR, 2010) OR LIMIT-T0 (PUBYEAR, 2009) OR LIMI T-TO (PUBYEAR, 2008) OR LI MIT-TO (PUBYEAR, 2007) OR LIMIT-TO (PUBYEAR, 2006) 0 R LIMIT-TO (PUBYEAR, 2005) OR LIMIT-TO (PUBYEAR, 200 4) OR LIMIT-TO (PUBYEAR, 2003) OR LIMIT-TO (PUBYEAR, 2002) OR LIMIT-TO (PUBY EAR, 2001) OR LIMIT-TO (PUBYEAR , 2000))	
hypertension AND (socio-cultural OR socioeconomic OR individual OR community OR "health system" OR "health financing") AND ("qualityofcare") AN D ("primary care" OR "primary healthcare") AND "West Africa" OR ghana OR niger OR "Burkina Faso" OR benin OR "Cape Verde" OR "Cote D'lvoire" OR gambia OR guinea OR "Guinea-Bissau" OR liberia OR mali OR mauritania OR nigeria OR senegal OR "Sierra Leon e" OR toga AND (LIMIT-TO(A FFILCOUNTRY, "Nigeria") OR LIMIT-TO (AFFILCOUNTRY , "Ghana") OR LIMIT-TO (AFFILCOUNTRY, "Benin") OR LIMIT-T0 (AFFILCOUNTRY, "Gambi a") OR LIMIT-TO (AFFILCOUNTRY, "Burkina Faso") OR LIMI T-TO (AFFILCOUNTRY, "Senegal") OR LIMIT-TO (AFFILCOUN TRY, "Liberia") OR LIMIT-T0 (AFFILCOUNTRY, "Sierra Leo ne") OR LIMIT-TO(AFFILCOUNTRY, "Cote d'lvoire") 0R LIMIT-TO(AFFILCOUNTRY, "Togo") OR LIMIT-TO (AFFILC OUNTRY, "Mali") OR LIMIT-T0 (AFFILCOUNTRY, "Niger") OR LIMIT-TO (AFFILCOUNTRY, "Cape Verde") OR LIMIT-T0 (AFFILCOUNTRY, "Guinea") OR LIMIT-TO (AFFILCOUNTRY, "Guinea-Bissau") OR LIMIT-TO (AFFILCOUNTRY, "Mauritania")) AND (LIMIT-TO(PUBYE AR, 2023) OR LIMIT-TO (PUB YEAR, 2022) OR LIMIT-TO (P UBYEAR, 2021) OR LIMIT-T0 (PUBYEAR, 2020) OR LIMI T-TO (PUBYEAR, 2019) OR LI MIT-TO (PUBYEAR, 2018) OR LIMIT-TO (PUBYEAR, 2017) 0 R LIMIT-TO (PUBYEAR, 2016) OR LIMIT-TO (PUBYEAR, 2015) OR LIMIT-TO (PUBYEAR, 2014) OR LIMIT-TO (PUBYEAR,2013) OR LIMIT-TO (PUBYEAR, 2012) OR LIMIT-TO (PUBYEAR, 2011) OR LIMIT-TO (PU BYEAR, 2010) OR LIMIT-T0 (PUBYEAR, 2009) OR LIMIT-TO (PUBYEAR, 2008) OR LIMIT-TO (PUBYEAR, 2007) OR LIMIT-TO (PUBYEAR, 2006) 0 R LIMIT-TO (PUBYEAR, 2005) OR LIMIT-TO (PUBYEAR, 2004) OR LIMIT-TO (PUBYEAR, 2003) OR LIMIT-TO (PUBYEAR, 2002) OR LIMIT-TO (PUBYEAR, 2001) OR LIMIT-TO (PUBYEAR , 2000))	
hypertension AND (socio-cultural OR socioeconomic OR individual OR community OR "health system" OR "health financing") AND (utilization OR	

For peer review only - http://bmjopen.bmj.com/site/about/guidelines.xhtml

access) AND ("primary care" OR "primary healthcare") AND "West Africa" OR ghana OR niger OR "Burkina Faso" OR benin OR "Cape Verde" OR "Cote D'lvoire" OR gambia OR guinea OR "Guinea-Bissau" OR liberia OR mali OR mauritania OR nigeria OR senegal OR "Sierra Leone" OR toga AND (LIMIT-T0 (AFFILCOUNTRY, "Nigeria") OR LIMIT-TO (AFFILCOUNTR Y, "Ghana") OR LIMIT-TO(AFFILCOUNTRY, "Benin") OR LI MIT-TO (AFFILCOUNTRY, "Gambia") OR LIMIT-TO (AFFILCOUNTRY, "Burkina Faso") OR LI MIT-TO (AFFILCOUNTRY, "Sen egal") OR LIMIT-TO (AFFILCO UNTRY, "Liberia") OR LIMIT-T0 (AFFILCOUNTRY, "Sierra Leo ne") OR LIMIT-TO(AFFILCOUNTRY, "Cote d'lvoire") 0R LIMIT-TO (AFFILCOUNTRY, "Togo") OR LIMIT-TO (AFFILC OUNTRY, "Mali") OR LIMIT-T0 (AFFILCOUNTRY, "Niger") OR LIMIT-TO (AFFILCOUNTRY, "CapeVerde") OR LIMIT T0 (AFFILCOUNTRY, "Guinea") OR LIMIT-TO (AFFILCOUNTRY, "Guinea-Bissau") OR LIMIT TO (AFFILCOUNTRY, "Maurita nia")) AND (LIMIT-TO (PUBYE AR, 2023) OR LIMIT-TO (PUB YEAR, 2022) OR LIMIT-TO (P UBYEAR, 2021) OR LIMIT-T0 (PUBYEAR, 2020) OR LIMI T-TO (PUBYEAR, 2019) OR LI MIT-TO (PUBYEAR, 2018) OR LIMIT-TO (PUBYEAR, 2017) 0 R LIMIT-TO (PUBYEAR, 2016) OR LIMIT-TO (PUBYEAR, 2015) OR LIMIT-TO (PUBYEAR, 2014) OR LIMIT-TO (PUBYEAR,<br>2013) OR LIMIT-TO (PUBYEAR, 2012) OR LIMIT-TO (PUBY EAR, 2011) OR LIMIT-TO (PUBYEAR, 2010) OR LIMIT-T0 (PUBYEAR, 2009) OR LIMI T-TO (PUBYEAR, 2008) OR LIMIT-TO (PUBYEAR, 2007) OR LIMIT-TO (PUBYEAR, 2006) 0 R LIMIT-TO (PUBYEAR, 2005) OR LIMIT-TO (PUBYEAR, 200 4) OR LIMIT-TO (PUBYEAR, 2003) OR LIMIT-TO (PUBYEAR, 2002) OR LIMIT-TO (PUBY EAR, 2001) OR LIMIT TO (PUBYEAR , 2000))	
Search query for Cairn Info	
1. "Soins primaire" ET "hypertension" ET "Afrique de l'Ouest"<br>2. "Accès" ET "Hypertension" ET "Afrique de l'Ouest"<br>3. "Accès" ET "Soins primaire" ET "hypertension"<br>4. Accès aux soins" ET "soins primaires" ET hypertension" ET "Afrique de l'ouest"<br>5. "Facteurs" ET "hypertension" ET " Afrique de l'Ouest"<br>6. "Facteurs" ET "soins primaires" ET "hypertension" ET " Afrique de l'Ouest"<br>7. "Influence" ET "hypertension" ET "Afrique de l'Ouest"<br>8. "Hypertension" ET "Afrique de l'Ouest"<br>9. "Utilisation" ET " Soins primaires" ET "hypertension" ET "Afrique de l'ouest"<br>10. "Qualité" ET " Soins primaires" ET "hypertension" ET "Afrique de l'ouest"	Lengthy combinations of key terms yielded zero results and as such, search terms were broken down into smaller combinations. After several varying combinations of key words, some papers were repeated in search results.

For peer review only - http://bmjopen.bmj.com/site/about/guidelines.xhtml

Appendix 3- PRISMA 2020 FLOW diagram for systematic reviews

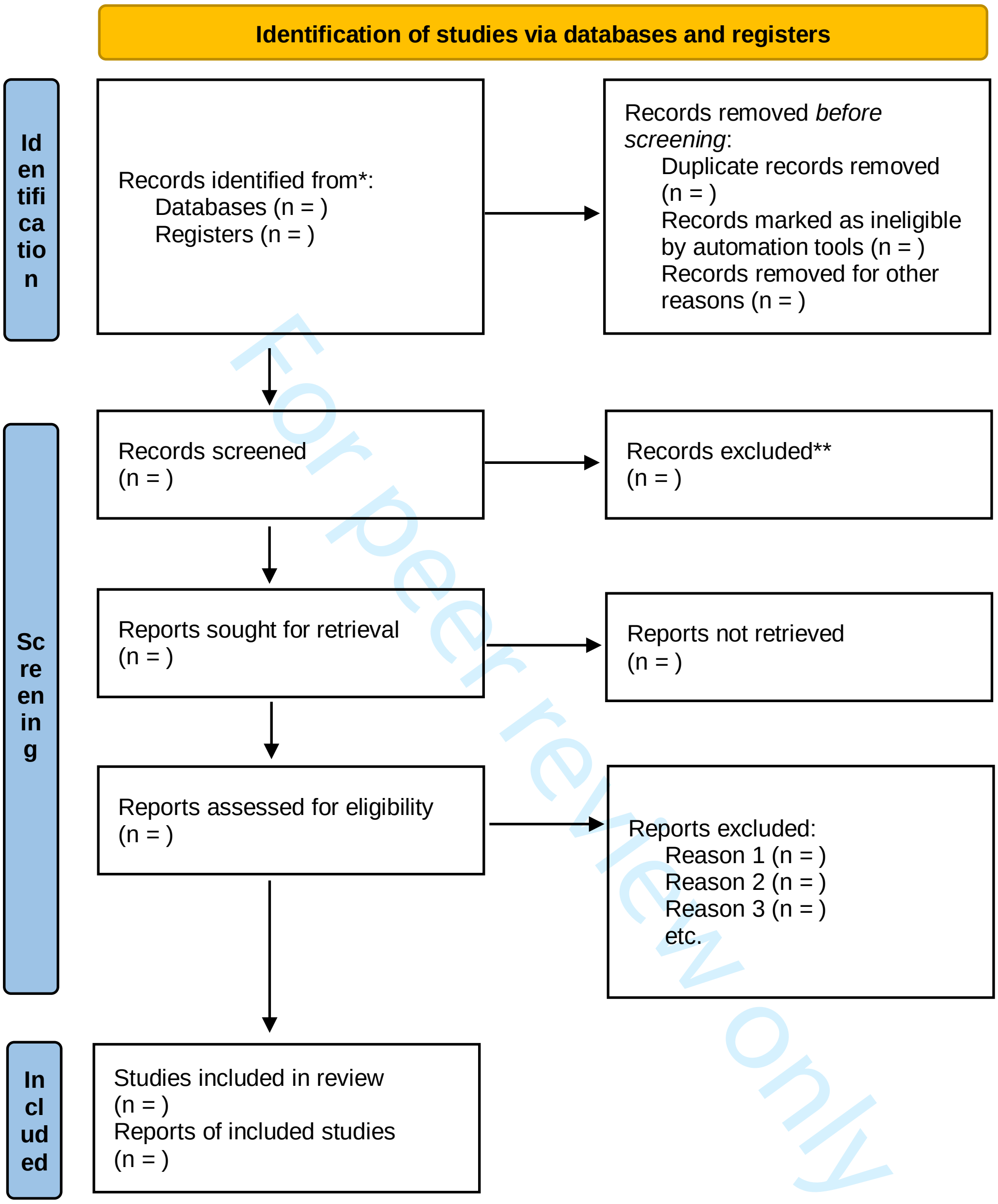

*Consider, if feasible to do so, reporting the number of records identified from each database or register searched (rather than the total number across all databases/registers).

**If automation tools were used, indicate how many records were excluded by a human and how many were excluded by automation tools.

For peer review only - http://bmjopen.bmj.com/site/about/guidelines.xhtml

Appendix 4- sample data charting form

Author (name and year)	Type of paper	Study design	Study population	Sample size	objectives	Key findings	type of evidence source	Comment/ limitations

For peer review only

For peer review only - http://bmjopen.bmj.com/site/about/guidelines.xhtml

Preferred Reporting Items for Systematic reviews and Meta-Analyses extension for Scoping Reviews (PRISMA-ScR) Checklist

SECTION	ITEM	PRISMA-ScR CHECKLIST ITEM	REPORTED ON PAGE #
**TITLE**			
Title	1	Identify the report as a scoping review.	
**ABSTRACT**			
Structured summary	2	Provide a structured summary that includes (as applicable): background, objectives, eligibility criteria, sources of evidence, charting methods, results, and conclusions that relate to the review questions and objectives.	
**INTRODUCTION**			
Rationale	3	Describe the rationale for the review in the context of what is already known. Explain why the review questions/objectives lend themselves to a scoping review approach.	
Objectives	4	Provide an explicit statement of the questions and objectives being addressed with reference to their key elements (e.g., population or participants, concepts, and context) or other relevant key elements used to conceptualize the review questions and/or objectives.	
**METHODS**			
Protocol and registration	5	Indicate whether a review protocol exists; state if and where it can be accessed (e.g., a Web address); and if available, provide registration information, including the registration number.	
Eligibility criteria	6	Specify characteristics of the sources of evidence used as eligibility criteria (e.g., years considered, language, and publication status), and provide a rationale.	
Information sources*	7	Describe all information sources in the search (e.g., databases with dates of coverage and contact with authors to identify additional sources), as well as the date the most recent search was executed.	
Search	8	Present the full electronic search strategy for at least 1 database, including any limits used, such that it could be repeated.	
Selection of sources of evidence†	9	State the process for selecting sources of evidence (i.e., screening and eligibility) included in the scoping review.	
Data charting process‡	10	Describe the methods of charting data from the included sources of evidence (e.g., calibrated forms or forms that have been tested by the team before their use, and whether data charting was done independently or in duplicate) and any processes for obtaining and confirming data from investigators.	
Data items	11	List and define all variables for which data were sought and any assumptions and simplifications made.	
Critical appraisal of individual sources of evidence§	12	If done, provide a rationale for conducting a critical appraisal of included sources of evidence; describe the methods used and how this information was used in any data synthesis (if appropriate).	
Synthesis of results	13	Describe the methods of handling and summarizing the data that were charted.	

For peer review only - http://bmjopen.bmj.com/site/about/guidelines.xhtml

SECTION	ITEM	PRISMA-ScR CHECKLIST ITEM	REPORTED ON PAGE #
**RESULTS**			
Selection of sources of evidence	14	Give numbers of sources of evidence screened, assessed for eligibility, and included in the review, with reasons for exclusions at each stage, ideally using a flow diagram.	
Characteristics of sources of evidence	15	For each source of evidence, present characteristics for which data were charted and provide the citations.	
Critical appraisal within sources of evidence	16	If done, present data on critical appraisal of included sources of evidence (see item 12).	
Results of individual sources of evidence	17	For each included source of evidence, present the relevant data that were charted that relate to the review questions and objectives.	
Synthesis of results	18	Summarize and/or present the charting results as they relate to the review questions and objectives.	
**DISCUSSION**			
Summary of evidence	19	Summarize the main results (including an overview of concepts, themes, and types of evidence available), link to the review questions and objectives, and consider the relevance to key groups.	
Limitations	20	Discuss the limitations of the scoping review process.	
Conclusions	21	Provide a general interpretation of the results with respect to the review questions and objectives, as well as potential implications and/or next steps.	
**FUNDING**			
Funding	22	Describe sources of funding for the included sources of evidence, as well as sources of funding for the scoping review. Describe the role of the funders of the scoping review.	

JBI = Joanna Briggs Institute; PRISMA-ScR = Preferred Reporting Items for Systematic reviews and Meta-Analyses extension for Scoping Reviews.

* Where *sources of evidence* (see second footnote) are compiled from, such as bibliographic databases, social media platforms, and Web sites.

† A more inclusive/heterogeneous term used to account for the different types of evidence or data sources (e.g., quantitative and/or qualitative research, expert opinion, and policy documents) that may be eligible in a scoping review as opposed to only studies. This is not to be confused with *information sources* (see first footnote).

‡ The frameworks by Arksey and O'Malley (6) and Levac and colleagues (7) and the JBI guidance (4, 5) refer to the process of data extraction in a scoping review as data charting.

§ The process of systematically examining research evidence to assess its validity, results, and relevance before using it to inform a decision. This term is used for items 12 and 19 instead of "risk of bias" (which is more applicable to systematic reviews of interventions) to include and acknowledge the various sources of evidence that may be used in a scoping review (e.g., quantitative and/or qualitative research, expert opinion, and policy document).

From: Tricco AC, Lillie E, Zarin W, O'Brien KK, Colquhoun H, Levac D, et al. PRISMA Extension for Scoping Reviews (PRISMAScR): Checklist and Explanation. Ann Intern Med. 2018;169:467–473. doi: 10.7326/M18-0850.

For peer review only - http://bmjopen.bmj.com/site/about/guidelines.xhtml
